# Global 0.25-degree gridded Snow water equivalent data derived from machine learning using *in-situ* measurements

**DOI:** 10.1038/s41597-026-06895-z

**Published:** 2026-03-10

**Authors:** Jungho Seo, Mahdi Panahi, JiHyun Kim, Sayed Bateni, Yeonjoo Kim

**Affiliations:** 1 Department of Civil and Environmental Engineering, Yonsei University, Seoul, 03722 South Korea; 2 Department of Civil, Environmental and Construction Engineering, and Water Resources Research Center, University of Hawaii at Manoa, Honolulu, HI 96822 USA; 3 Department of Geography, Kyung Hee University, Seoul, 02447 South Korea; 4 Department of Climate-Social Science Convergence, Kyung Hee University, Seoul, 02447 South Korea; 5 UNESCO-UNISA African Chair in Nanoscience and Nanotechnology College of Graduate Studies, University of South Africa, Muckleneuk Ridge, Pretoria, 392 South Africa

**Keywords:** Hydrology, Hydrology

## Abstract

In this study, we developed a machine learning-based global daily SWE product (SWEML) with 0.25° (~25 km) resolution for 1980–2020. Using k-means clustering, *in-situ* SWE measurements were grouped into 14 clusters, and a random forest model was trained on 11,687 grid points with meteorological forcing and terrain attributes. SWEML was compared with ten reference datasets representing diverse approaches, including land–atmosphere reanalysis without data assimilation (DA), systems incorporating DA, snow model simulations of varying complexity driven by reanalysis forcing with or without DA, and remote sensing products. The overall root mean square error (RMSE) and bias were 10.33 mm and −7.13 mm, respectively. Notably, SWEML achieved high accuracy in high-elevation regions such as the Rocky Mountains, with an RMSE of 7.30 mm and correlation coefficient of 0.98. It also agreed with the Gamma airborne SWE over North America and showed similar spatial patterns and peak SWE time series of the Andes Snow Reanalysis. These results highlight the robustness of SWEML in regions with and without training data.

## Background & Summary

Snow is a vital component of Earth’s hydrological cycle, influencing the volume of water flowing into rivers, lakes, and streams and serving as an important water source for millions of people^1–3^. Snow is commonly characterized by its depth, cover, and snow water equivalent (SWE), which quantifies the amount of water stored within the snowpack. Accurate estimation of SWE is essential for a wide range of applications, including water resource management^3^, global water cycle modeling^4^, and the prediction of natural hazards such as landslides^5^, floods^6^, and droughts^7^. Despite being recognized as an essential climate variable by the Global Climate Observing System (GCOS) of the World Meteorological Organization, SWE remains one of the least observed components of the global water cycle^8–10^.

Several ground-based observation networks have been established to measure SWE, including the SNOwpack TELemetry Network (SNOTEL), the Russian Route Snow Survey Data (RSSD), the Global Historical Climatology Network daily (GHCNd), and the Norwegian Water Resources and Energy Directorate (NVE). However, these ground-based measurements are costly, labor-intensive, limited to point-scale data, making them insufficient to represent the broad spatial coverage of the Northern Hemisphere^10^. As an alternative, passive remote sensing observations provide long-term SWE records at the global scale^9–11^. Nevertheless, SWE retrievals from remote sensing are subject to inherent limitations depending on the sensors mounted on each instrument. For example, SWE retrievals from passive microwave instruments such as the Special Sensor Microwave/Imager (SSM/I) and the Advanced Microwave Scanning Radiometer for Earth Observing System (AMSR-E) show large uncertainties in deep snow conditions (i.e., SWE >150 mm) and are highly sensitive to forest cover^9,11,12^. Therefore, considerable efforts have been directed toward utilizing radar data across multiple bands (e.g., C, P, Ku, and X) to generate spatially distributed SWE datasets with the potential for long-term monitoring^13^. Although active radar data have shown promising results, their operational use remains challenging and requires advanced technical developments^10,14^.

In addition to passive remote sensing observations, physically based models also provide global and regional SWE estimates^15^. However, their outputs often differ substantially due to uncertainties in model parameterizations, simplified assumptions, and structural limitations^16–19^. To address these challenges, Pulliainen *et al*.^20^ and Takala *et al*.^21^ developed the GlobSnow SWE dataset by combining ground-based synoptic weather measurements with spaceborne passive microwave data using a Bayesian non-linear iterative assimilation approach. Despite certain limitations, such as overestimation and the exclusion of mountain regions, GlobSnow v3.0 (ESAGB) remains a reliable SWE product at the continental scale across the Northern Hemisphere^22^. Each existing dataset has its own unique limitations. Therefore, there is still a pressing need to develop an accurate SWE product that can be reliably used by the scientific community^12,23^.

Machine learning (ML) has recently garnered increasing attention as a viable alternative for integrating independent variables from diverse sources to estimate SWE^24–27^. ML approaches have proven effective at capturing the complex relationships between meteorological variables (i.e., predictors) and SWE^26^. Consequently, numerous studies have applied ML techniques to SWE estimation. For instance, Broxton *et al*.^24^ employed an artificial neural network to integrate snow density measurements with lidar-derived snow depth data, thereby generating high-resolution SWE maps. Shao *et al*.^25^ applied a ridge regression algorithm to estimate SWE in regions north of 45°N, while Cui *et al*.^26^ used a long short-term memory algorithm to estimate SWE in the Sierra Nevada, California. These studies demonstrate that ML algorithms can provide more accurate SWE estimates than traditional physics-based or remote sensing approaches^24–27^ In addition to SWE estimation, ML techniques have also been successfully applied to predict or simulate various hydrometeorological processes, including runoff^28^, streamflow^29,30^, soil moisture^31,32^, and precipitation^33^, as well as their associated social impacts^34^. These applications leverage diverse meteorological and geographical datasets spanning local to global scales.

**Fig. 1:**
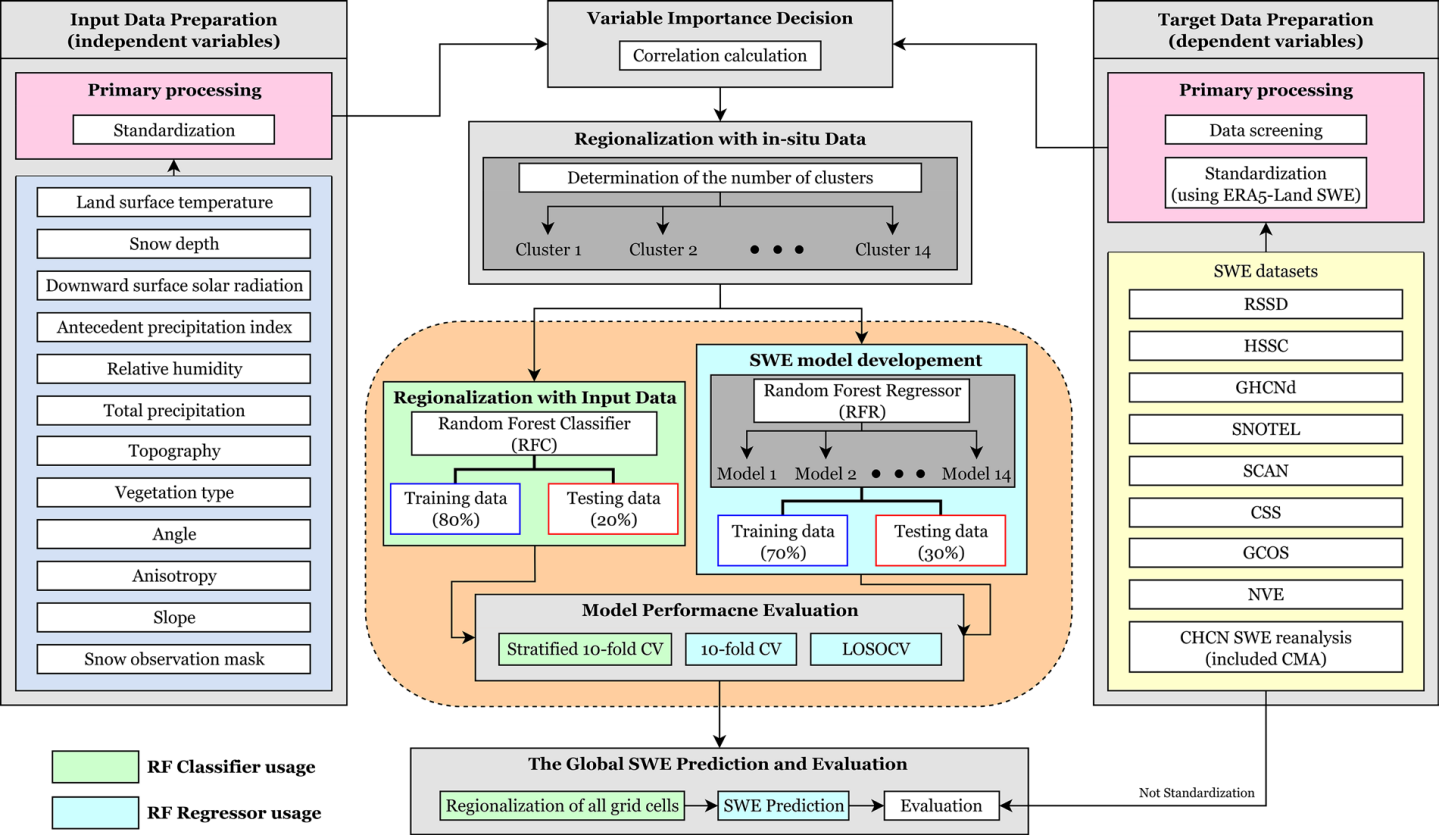
Schematic overview of the workflow for obtaining the RF-based global gridded SWE (SWEML) product. Point-scale *in-situ* SWE measurements are first allocated to 0.25° grid cells using the long-term mean and standard deviation of SWE from ERA5-Land. These grid cells are then grouped into 14 clusters through Random Forest classification. Within each cluster, a Random Forest regression model is applied to predict SWE. The resulting global SWE estimates are validated against *in-situ* measurements and compared with reference datasets.

In this study, we developed a new global-scale SWE product for the 1980–2020 period using the random forest (RF) algorithm. The RF algorithm employs meteorological and terrain attributes as predictor variables and relates them to *in-situ* SWE measurements as the dependent variable. To build the SWE estimation framework, we collected *in-situ* measurements from diverse sources along with reanalysis data spanning 1980–2020. We then applied mini-batch k-means (MBK) clustering to regionalize these measurements. Based on this, we constructed SWEML, a global daily SWE product covering all land areas except Antarctica, using RF models trained separately for each cluster. SWEML was evaluated against multiple reference datasets and demonstrated improved performance, particularly in high-elevation regions, as well as an enhanced ability to capture monthly trends reflected in *in-situ* measurements. Region-specific evaluations using high-resolution and spatially representative reference datasets further demonstrated the robustness of SWEML, particularly in the Andes, where no training data were available, as well as across North America. A schematic overview of the study design is shown in Fig. 1.

## Methods

### SWE data

#### *In-situ* measurements and remote sensing data

**Fig. 2:**
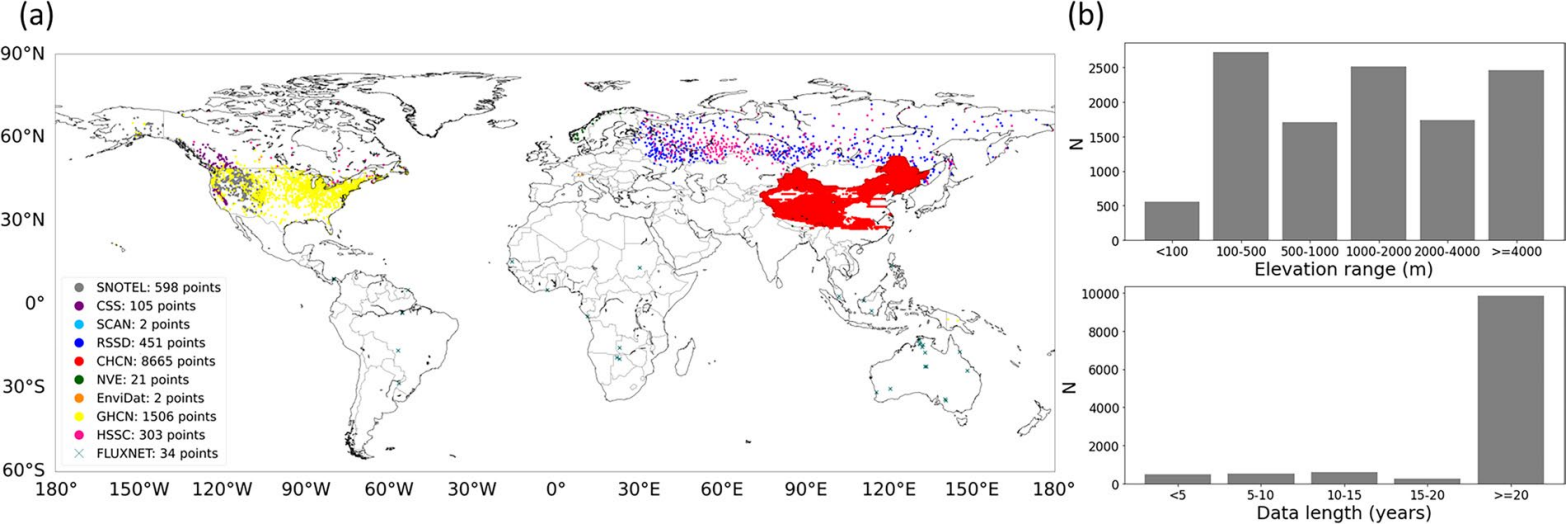
(**a**) Spatial distribution of the *in-situ* SWE measurements used in this study (11,687 grid points). (**b**) Distribution of grid cells by elevation and data availability during the study period (1980–2020).

In this study, we collected two types of daily *in-situ* SWE measurements (snow course and automated sensors) from eight different sources across the Northern Hemisphere (Table 1 and Fig. 2a). We used SWE data from 517 snow course sites in Russia provided by the RSSD, obtained from the All-Russia Research Institute of Hydrometeorological Information – World Data Center^35^. The RSSD surveys are conducted every 10 days during the cold season and every five days during the snowmelt period, with measurements performed separately for three land cover types (field, forest, and gully)^36^. When SWE data from multiple land cover types were available for the same date, we averaged them for use in this study. We also used 3,075 SWE data points from the Hemispheric-Scale Snow Course (HSSC) dataset, a hemispheric-scale database developed by Pulliainen *et al*.^9^ using snow course observations from the former Soviet Union/Russia (FSU), Finland, and Canada^37,38^. The HSSC provides transect-based observations, where SWE is manually measured at multiple locations under representative snow and land cover conditions, reducing uncertainty in regional-scale SWE variability associated with snowpack properties and surface heterogeneity^9^.

**Table 1:** List of SWE data sources used in this study for model training and data rescaling.

Data type	Dataset/Product	Data period*	Resolution		Spatial coverage
			Temporal	Spatial	
*In-situ*	RSSD (Russia Research Institute of Hydrometeorological Information; RIHMI-WDC)	1980-01 to 2020-12	5–15 daily	517 sites	Russia
	HSSC (Finnish Meteorological Institute; FMI)	1980-01 to 2018-04	5–15 daily	3,075 data points	Canada, Finland, and former Soviet Union/Russia (FSU)
	GHCNd (National Oceanic and Atmospheric Administration; NOAA)	1981-01 to 2020-12	Daily	15,031 data points	CONUS
	SNOTEL (USDA NRCS)	1980-01 to 2020-12	Daily	848 sites	Western U.S., Alaska
	SCAN (USDA NRCS)	1994-10 to 2020-12		2 sites	Mideast U.S.
	CSS (USDA NRCS)	1980-01 to 2020-12		144 sites	Western U.S, Canada
	GCOS (EnviDat)	1980-01 to 2020-12	15 daily	11 sites	Switzerland
	NVE (Norwegian Water Resources and Energy Directorate)	1980-01 to 2020-12	Daily	21 sites	Norway, Nepal
	FLUXNET Precipitation Data using a Temperature threshold	1980-01 to 2020-12	Daily	34 sites	S.America, Australia, Africa, South Asia
Remote Sensing (bias-corrected)	Daily SWE Dataset^54^ [including *in-situ* measurements from the China Meteorological Administration (CMA)]	1980-01 to 2020-12	1–5 daily	25 km	China
Reanalysis	ERA5-Land SWE	1980-01 to 2020-12	Hourly	10 km	Global

*The listed data periods represent the maximum available time span for each dataset or product used in this study.

The GHCNd provides daily climate records from ground stations in 180 countries and territories, which have been integrated and subjected to standardized quality assurance checks^39,40^. Among its parameters, the dataset includes SWE measurements from various contributing networks, listed under the WESD variable^41^. For this study, we used 15,031 GHCNd SWE data points exclusively from the United States, owing to temporal limitations, and excluded SNOTEL data to avoid duplication. The SNOTEL network is an extensive system of automated stations that measure SWE using snow pillows, which weigh the water content of accumulated snow. It spans the western U.S., including high-latitude tundra and taiga regions in Alaska, the Rocky Mountains, and the maritime Cascade Range^42–44^. SNOTEL only includes stations where an average of at least 40 days of continuous snow cover is observed. In this study, we used SWE data from 848 SNOTEL sites. The Soil Climate Analysis Network (SCAN) is another automated system that primarily monitors soil moisture and climate conditions^43,45^. SCAN provides SWE data at only two sites, located in the north-central and northeastern U.S.^46^. We included SWE data from both sites in our study^47^. The Cooperator Snow Sensors (CSS) network, managed by individual state or provincial agencies in California, British Columbia, and Alberta, also provides automated SWE data from snow pillows^48,49^. SWE data were collected from 144 CSS sites via the USDA Natural Resources Conservation Service Interactive Map^47^. Additionally, we included real-time, quality-checked SWE measurements from 21 sites maintained by NVE^50,51^, as well as biweekly manual SWE measurements from 11 Swiss sites provided by GCOS^52^. Most of the collected *in-situ* measurements are concentrated in Russia, Europe, and North America. However, the Tibetan Plateau, where snow plays a critical role in the hydrological cycle^53^, remains poorly represented in global datasets due to the inherent difficulties of direct data collection. To compensate for this, we used bias-corrected remote sensing data for the SWE in China with a spatial resolution of 0.25°. These data incorporate passive microwave observations with *in-situ* measurements and snow course surveys from the National Meteorological Information Centre of the China Meteorological Administration^54^.

In regions with tropical and hot-dry climates as defined by the Köppen climate classification, snowfall is rarely observed. To account for these areas where snow accumulation does not occur, we used precipitation and temperature data from 34 FLUXNET sites and screened snowfall events using a temperature threshold^55,56^. We set this threshold at 4 °C, above which snowfall frequency typically approaches zero^57^. Although the precipitation phase threshold is highly sensitive to relative humidity, studies have shown that snowfall frequency decreases sharply beyond 4 °C when relative humidity exceeds 70%^58^, a common condition in tropical regions.

#### Reference datasets for validation

In this study, we selected three types of reference datasets to validate SWEML (Table 2). These products represent diverse approaches, including land–atmosphere reanalysis without data assimilation, systems that incorporate data assimilation techniques, snow model simulations of varying complexity driven by reanalysis forcing (with and without data assimilation), and remote sensing. All reference SWE products and hyperlinks to all associated data are provided in Table 3.

**Table 2:** List of reference SWE data sources used in this study.

Data type	Datasets/products	Method	Data periods	Resolution		Domain availability
				Temporal	Spatial	
Remote sensing	AMSR-E/AMSR2 (Aqua/GCOM-W1)	Standalone passive microwave (PMW)	2003-01 to 2020-12 (excluding 2011 through 2012)	Daily	0.25°	Global (except Greenland)
	GAMMA	Airborne Gamma Survey	1980-01 to 2020-12		point	CONUS/Transboundary Canadian watersheds
Model	Brown-ERA5	Temperature index snow model + ERA5 forcing	1981-01 to 2020-12		0.25°	NH above 10° latitude
	CROCUS-ERA5	Crocus snow model + ERA5 forcing	1980-01 to 2020-12			NH above 25° latitude
Reanalysis	GLDAS v2.0	Reanalysis (Catchment LSM)	1980-01 to 2003-01		0.25°	Global
	GLDAS v2.2	Reanalysis (Catchment LSM) + Total terrestrial water anomaly assimilation (GRACE)	2003-02 to 2020-12			
	Andes-SR	SSiB-SDC + Lansat fSCA (PBS) assimilation	1985-01 to 2015-12		6 arcsec (~180 m)	Andes
	MERRA-2	Reanalysis (Catchment LSM)	1980-01 to 2020-12	Hourly	0.5° × 0.625°	Global
Assimilation	ESA GlobSnow v3	Passive microwave + snow depth (SD) assimilation	1980-01 to 2020-12	Daily	0.25°	North Hemisphere above 35° latitude (except Greenland)
	ESA CCI CDR v3.1		1980-01 to 2020-12		0.1°	NH (except mountain area)
	U. Arizona	PRISM + SWE and SD assimilation (SNOTEL, COOP)	1981-01 to 2020-12		0.04° (~4 km)	CONUS

**Table 3:** Availability of the SWE products and measurements used in this study.

Product name	Source/DOI
NASA-AMSR-E^77^ JAXA-AMSR2^79^	10.5067/AMSR-E/AE_DYSNO.002 10.5067/8AE2ILXB5SM6
B-TIM-ERA5^74^	10.5683/SP3/HHIRBU
Crocus-ERA5^75^	10.5281/zenodo.10943718
MERRA-2^67^	10.5067/RKPHT8KC1Y1T
ESA-SnowCCIv3.1^70^ ESA-GlobSnow v3^22^	https://catalogue.ceda.ac.uk/uuid/9d9bfc488ec54b1297eca2c9662f9c81 https://www.globsnow.info/swe
GLDAS v2.2 CLSM^63^ GLDAS v2.0 CLSM^60^	10.5067/TXBMLX370XX8 10.5067/LYHA9088MFWQ
U.Arizona^81^	10.5067/0GGPB220EX6A
Andes-SR^138^	10.5061/dryad.ngf1vhj0s
ERA5^95^ ERA5-Land^139^	10.24381/cds.adbb2d47 10.24381/cds.e2161bac
CSS-SR-China^140^	10.11922/sciencedb.j00076.00071
GAMMA^83^	https://www.nohrsc.noaa.gov/snowsurvey (last access: March 1, 2025)
HSSC^9^	https://www.globsnow.info/swe/archive_v3.0/auxiliary_data
RSSD^35^	http://aisori-m.meteo.ru/waisori/index0.xhtml (last access: March 1, 2025)
SNOTEL^44^ SCAN^45^ CSS^47^	https://nwcc-apps.sc.egov.usda.gov/imap (last access: March 1, 2025)
GCOS^52^	https://doi.org/10.16904/15
GHCN^39^,^40^	10.7289/V5D21VHZ
NVE^51^	https://sildre.nve.no/list?paramsStationList (last access: March 1, 2025)

The Global Land Data Assimilation System (GLDAS) integrates a wide range of observation-based data to drive multiple land surface models in an uncoupled framework, producing globally gridded land surface variables^59^. For this study, we used SWE datasets from the GLDAS Catchment Land Surface Model (CLSM) version 2.0^60,61^ and version 2.2^62,63^. Both versions are forced by the Princeton meteorological forcing dataset. However, version 2.2 additionally incorporates GRACE data assimilation, which is not included in version 2.0. We also used SWE from the Modern-Era Retrospective Analysis for Research and Applications Version 2 (MERRA-2), an atmospheric reanalysis dataset of the satellite era produced by the NASA Global Modeling and Assimilation Office using the Goddard Earth Observing System Model version 5.12.4^64^. In MERRA-2, land surface processes are parameterized using CLSM^65,66^. The SWE dataset provided by MERRA-2^67^ was employed in this study.

Additionally, the ESAGB dataset provides SWE and snow-cover data for the Northern Hemisphere north of 35°N, released by the European Space Agency (ESA)^22^. Its SWE estimates combine Canadian Meteorological Center daily snow depth analyses^68^, ground-based weather station data, and satellite-based microwave radiometer observations. ESA also offers the long-term Snow Climate Change Initiative (CCI+) dataset family, which follows a methodology similar to the GlobSnow algorithm. In version 2, updated passive microwave data from the NASA Measures Calibrated Passive Microwave Daily EASE-Grid 2.0 Earth Science Data Record were used, and the grid spacing was refined from 25 km to 12.5 km. Additionally, SWE retrievals were adjusted during post-processing by incorporating spatially and temporally varying snow density information, thereby improving accuracy^69^. More recently, the Snow CCI + SWE version 3.1 (ESASWE) was released, introducing improved dry-snow detection and a refined snow mask for post-processing^70^. However, both ESAGB and ESASWE products have limited spatial coverage, excluding regions with complex terrain and areas below 35°N and the Equator, respectively.

The Brown Temperature Index Model (B-TIM; BROWN) and the Crocus snowpack model (CROCUS) are physically based models that provide distinct families of products depending on the meteorological forcing applied. Each model relies on a different snow scheme: a simple temperature index scheme in the case of B-TIM^71,72^, and a complex multilayer snow scheme embedded within the Interactions between Soil–Biosphere–Atmosphere (ISBA) land surface model in the case of Crocus^73^. In this study, we used SWE datasets from the forcing-based B-TIM^74^ and Crocus products^75^, both derived from the ERA5 reanalysis forcing, which has been identified as one of the best-performing datasets^76^.

The AMSR-E/AMSR2 dataset is satellite-based and derives from microwave scanning radiometers onboard NASA’s Aqua satellite (Earth Observation System) and the GCOM-W1 satellite of the Japan Aerospace Exploration Agency (JAXA). The AMSR-E sensor aboard Aqua provided daily SWE data from 19 June 2002 to 3 October 2011^77,78^, while the AMSR2 sensor aboard GCOM-W1 has been providing daily SWE data since 2 July 2012^79,80^. For this study, we used SWE data with a spatial resolution of 0.25° (approximately 25 km) and a daily temporal resolution. Although AMSR-E/AMSR2 observations began in 2002, there are gaps between October 2011 and June 2012.

Most gridded SWE datasets have a spatial resolution of 0.25° (approximately 25 km), which covers continental scales such as the part of the Northern Hemisphere or the globe. However, this spatial resolution may be insufficient for accurately representing SWE in mountainous regions with complex topography, such as the Andes in the Southern Hemisphere. To address this limitation, we used two additional high-resolution SWE datasets and one airborne observation dataset: the University of Arizona SWE (UASWE)^81^, the Andes Snow Reanalysis SWE (ADSWE)^82^, and the Airborne Gamma SWE (GAMMA)^83^.

UASWE was developed by assimilating *in-situ* SWE and snow depth measurements with precipitation and temperature data from the Parameter-elevation Regressions on Independent Slopes Model (PRISM) across the Continental United States^84,85^. These *in-situ* measurements include SWE and snow depth from SNOTEL stations, as well as snow depth from the NOAA National Weather Service Cooperative Observer Program (COOP). COOP snow depth data were converted to SWE using a newly developed snow density parameterization^86^, allowing for the consistent integration of SNOTEL and COOP observations into a unified SWE dataset. ADSWE was developed using a Bayesian framework^87^ that assimilates fractional snow-covered area images derived from Landsat into the SSiB3 land surface model with a snow depletion curve, using a particle batch smoother^82,88^. Finally, GAMMA, operated by the National Operational Hydrologic Remote Sensing Center (NOHRSC) snow survey program, provides near-real-time areal SWE estimates across the United States and portions of the Canadian provinces. These areal SWE estimates are calculated as spatial averages over each flight line footprint, typically 15–20 km in length and ~300 m in width, covering an area of approximately 5 km^2^ ^83^. Therefore, these estimates reliably represent SWE at the relevant spatial scales and have been widely used to evaluate gridded SWE products in regions of the United States with available historical records^89^. The GAMMA approach estimates SWE by measuring the attenuation of naturally emitted gamma-ray signals, based on the difference in gamma radioisotope intensities between snow-covered and snow-free land surfaces^46,83^.

### Ancillary data

#### Topographical data

Five terrain characteristics, including vegetation type, elevation, slope, aspect, and anisotropy of the orography, were collected from multiple sources (Table 4) and used as input variables for the RF algorithm due to their significant influence on SWE. Vegetation type data were obtained from the GLDAS Dominant Vegetation Type Dataset Version 2. Vegetation affects snow accumulation and melt through shading effects, such as canopy interception and changes in the radiation balance, which can influence ablation by up to 70% and accumulation by up to 40% compared to nearby open areas^90^.

Elevation is a key predictor of SWE and has been widely used in previous studies^15,25^. Higher elevations generally correspond to lower temperatures, resulting in an earlier onset of the snow season and delayed melting compared to lower elevations^91^. Elevation also interacts with land cover to influence snow accumulation, with more pronounced differences in melting observed between clear-cut and forested areas at higher elevations^92,93^. For this study, we used the mean and standard deviation of digital elevation data obtained from ETOPO1^94^.

For orographic variables, which describe terrain features in mountainous regions, we used slope, aspect, and anisotropy derived from the ERA5 reanalysis dataset^95^. ERA5 provides a realistic representation of orographic variability across diverse terrains^96^. These variables are closely related to solar radiation and wind effects, both of which significantly influence snow distribution in mountainous areas^93^. Consequently, numerous studies have adopted these variables as predictors of SWE^26,97^.

**Fig. 3:**
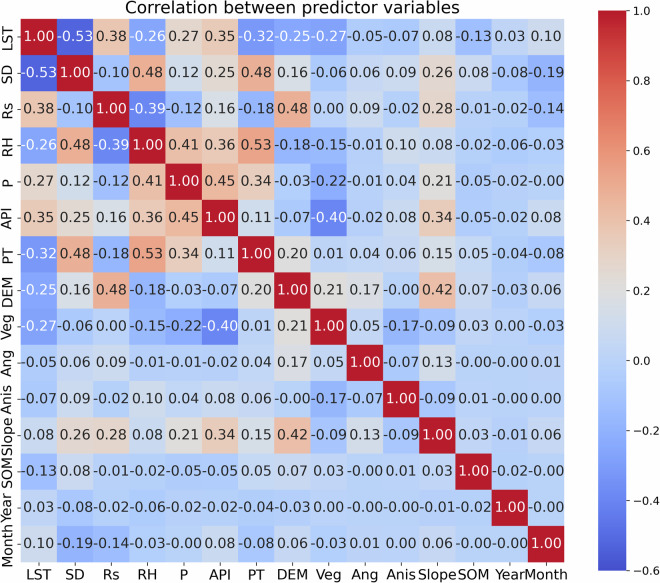
Correlation matrix showing the relationships between input predictors: LST (land surface temperature), SD (snow depth), Rs (downward solar radiation), RH (relative humidity), P (precipitation), API (antecedent precipitation index), PT (precipitation type), DEM (digital elevation map), Veg (vegetation type), Anis (anisotropy of orography), Slope (slope of orography), and SOM (snow observation mask).

Elevation and the orographic variables, including slope, aspect, and anisotropy, were identified as highly influential predictors of model performance based on variable importance and sensitivity analyses using permutation variable importance (PVI) and Sobol sensitivity analysis (Fig. S1). Among the orographic variables, slope exhibited a positive correlation of 0.42 with elevation (Fig. 3). Despite this correlation, permutation importance indicated that perturbing either elevation or slope led to a degradation in SWE estimation skill, while the Sobol analysis demonstrated that both variables retained substantial independent and interaction-based contributions to SWE variability (Fig. S1b). These results demonstrate that elevation and slope provided independent and complementary predictive information even at the 0.25° resolution.

#### Meteorological data

We used hourly meteorological forcing data with a spatial resolution of 0.25° (approximately 25 km) from the ERA5 reanalysis dataset, provided by ECMWF^95^. We converted the temporal resolution of meteorological forcing data from hourly to daily for use in this study. The ERA5 reanalysis dataset integrates diverse data sources, including ground-based synoptic stations, radar precipitation measurements, and satellite radiances, via 4D-Var data assimilation to produce comprehensive and accurate reanalysis products^98^. Compared to its predecessor, ERA-Interim, ERA5 incorporates more observational data and advanced modeling techniques, providing improved meteorological forcing data^99^. Furthermore, ERA5 reanalysis dataset provides a consistent data structure suitable for generating long-term global-scale datasets. Direct use of observational data, such as satellite measurements, can present challenges due to spatiotemporal inconsistencies between variables of interest.

In this study, the RF model was trained using six meteorological forcings—precipitation, precipitation type, relative humidity, snow depth, land surface temperature, and downward solar radiation—along with the antecedent precipitation index (API), temporal indicators (year and month), and a snow observation mask (Table 4 and Fig. S2). Temporal indicators were also included to account for interannual and seasonal variability. We selected the input forcing variables based on a literature review and correlation analysis. As shown in Fig. 3, each input variable exhibited weak correlations with other inputs, indicating that they can be considered largely independent. Among the selected forcings, snow depth exhibits the strongest negative correlation with land surface temperature, while relative humidity shows the strongest positive correlation with precipitation type. Relative humidity, in particular, influences the temperature threshold for rain–snow partitioning^58,100,101^. Precipitation is a fundamental factor controlling SWE^102–104^, as SWE is sensitive both to the total amount of precipitation^84,104^ and to rain–snow partitioning^105^. Therefore, precipitation type (rain or snow) over land^95^ and relative humidity were included as input variables to account for rain–snow partitioning. Snow depth directly affects SWE and was thus included as a predictor^106^. Snow depth was consistently highly ranked in terms of variable importance, permutation sensitivity, and both first-order and total-order Sobol indices, indicating strong direct and interaction-driven influences on SWE estimation (Fig. S1). Land surface temperature and downward solar radiation influence snow phase transitions by altering melt and freeze behaviors upon contact with the surface, and consequently have a significant impact on SWE^107,108^. Additionally, we included the API, which represents accumulated precipitation, as an input variable due to the influence of prior-day precipitation on SWE. The API is calculated as the weighted sum of daily precipitation over previous days^109,110^ and is defined as follows:

1 $${API}=\mathop{\sum }\limits_{n=-1}^{-i}{P}_{n}{k}^{-n},$$

where ${P}_{n}$ is the precipitation on the *n*th day, $i$ denotes the number of previous days, and $k$ is the decay constant. Typically, $i$ is set to 5 days or more^111,112^, while $k$, an empirical parameter, is assigned values ranging from 0.8 to 1.0^113–116^. In this study, *k* was varied from 0.8 to 1 in increments of 0.05, while *i* varied from 1 to 30 in increments of 1. The values of $i$ and *k* that yielded the highest correlation between the API and *in-situ* SWE were selected. Specifically, when $i$ and *k* were set to 23 and 1, respectively, the model produced the strongest correlation coefficient between API and SWE. Additionally, we included a snow observation mask to account for tropical regions where snow is rarely observed. The snow observation mask was generated using ERA5-Land SWE, which best represents the trend and variability of SWE^76^, together with snow depth and precipitation type variables from the ERA5 reanalysis dataset. For each grid cell over the study period, the mask was assigned a value of 1 if snow was observed and 0 if not (Fig. S2).

**Table 4:** Input data used in the RF model.

	Variable	Source	Description
Dynamic	Precipitation (P)	ERA5	Daily meteorological forcing data obtained from the ECMWF reanalysis
	Precipitation type (PT)		
	Relative humidity (RH)		
	Snow depth (SD)		
	Land surface temperature (LST)		
	Downward solar radiation (Rs)		
	Antecedent precipitation index (API)		Calculated from precipitation measurements on the given day and preceding days
	Year		Year of *in-situ* SWE measurement
	Month		Month of *in-situ* SWE measurement
Invariant	Vegetation type (Veg)	GLDAS (https://ldas.gsfc.nasa.gov/gldas/vegetation-class-mask)	Predominant vegetation type within each grid cell (MODIS-derived)
	Digital elevation map (DEM)	ETOPO1	Mean and standard deviation of sub-grid scale elevation values within each grid cell
	Angle of orography (Ang)	ERA5	Mean and standard deviation of sub-grid scale orography angle values within each grid cell
	Anisotropy of orography (Anis)		Mean and standard deviation of sub-grid scale orography anisotropy values within each grid cell
	Slope of orography (Slope)		Mean and standard deviation of sub-grid scale slope values within each grid cell
	Snow observation mask (SOM)	ERA5 and ERA5-Land	Derived using ERA5-Land SWE, snow depth, and precipitation type to create a binary snow mask

#### Data screening and rescaling

To ensure data quality, we screened the *in-situ* measurements (RSSD, HSSC, GHCNd, SNOTEL, SCAN, CSS, GCOS, and NVE) used for training, validation, and testing. Data points and sites with fewer than one month of observations were excluded, and null or negative values were removed. Additionally, a decimal error was corrected, and extreme SWE values exceeding 2 m were defined as outliers and removed. Using the coordinates of each point-scale *in-situ* measurement, we assigned each observation to the corresponding 0.25° grid cell defined by the ancillary dataset. When multiple measurements, either of the same type or different types were assigned to the same grid cell, their average was used. If data points representing SWE on different dates were assigned to the same grid cell, all were used. Grid cells with less than three months of SWE data were discarded. In total, we collected SWE data at 11,687 grid points, with most covering a period of more than 20 years and having a consistent elevation distribution (Fig. 2b).

*In-situ* measurements collected by various agencies and countries lack consistency due to differences in calibration. Therefore, variations in the mean and variance across time series can introduce artifacts and affect the reliability of ML training. Furthermore, point-scale measurements may lack spatial representativeness, particularly in regions with high spatial heterogeneity such as high-elevation areas. To address these issues, we rescaled the raw *in-situ* SWE measurements within each grid cell to match the mean and variance of the corresponding ERA5-Land SWE data provided by the European Centre for Medium-Range Weather Forecasts (ECMWF). As a result, the rescaled *in-situ* measurements resemble ERA5-Land SWE in terms of mean and standard deviation, enabling the consistent use of data from different sites and time periods while preserving the temporal variability observed in the *in-situ* measurements. Notably, SWE information from *in-situ* measurements is particularly effective at capturing temporal variability and physical snow processes^117^. Based on this, we assume that *in-situ* measurements provide sufficient information to infer SWE dynamics at the grid scale.

#### Regionalization

**Fig. 4:**
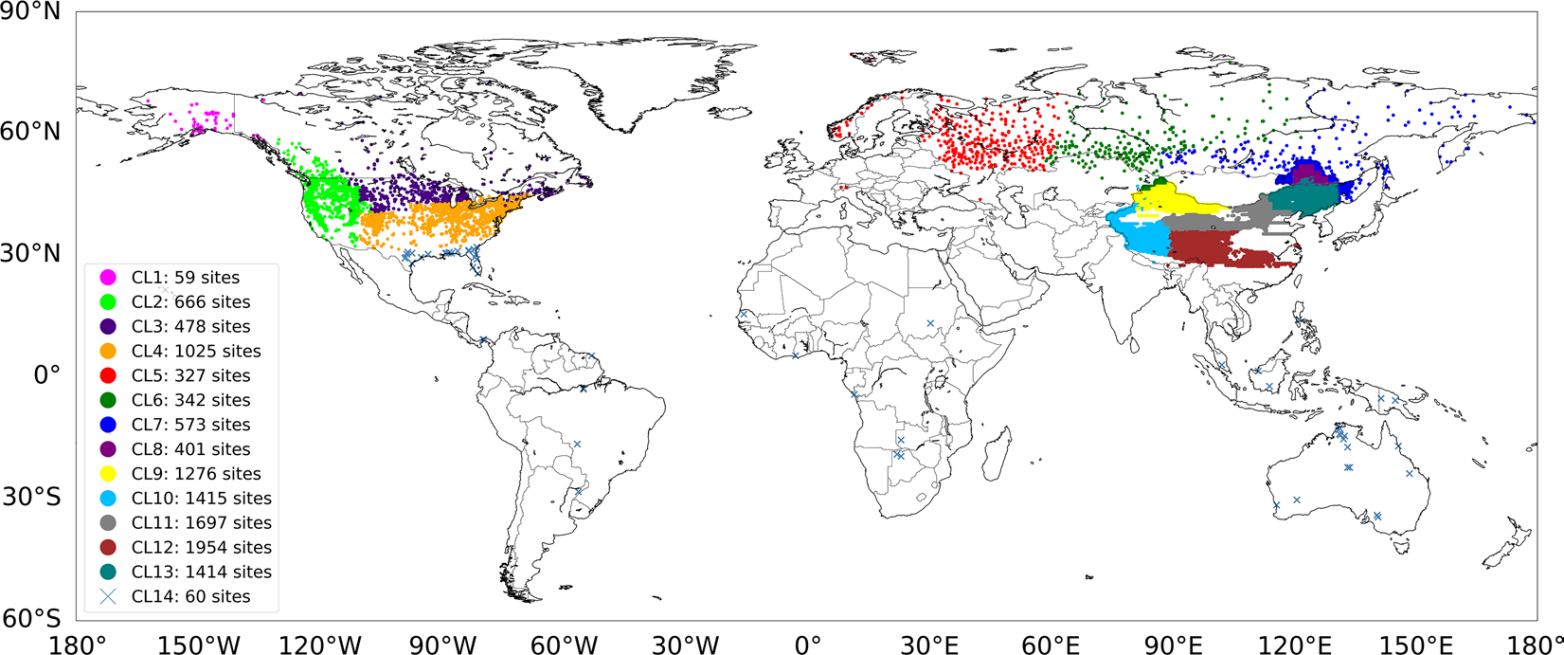
Regionalization map produced using MiniBatch K-means (MBK) clustering, showing the number of *in-situ* measurement sites within each cluster (CL).

SWE exhibits regional variability due to meteorological and geographical characteristics^9,76^. Consequently, regionalization is a useful approach for analyzing SWE dynamics, especially when data are collected across different continents with diverse meteorological and geographical characteristics^118^. In this study, we regionalized the training and testing data (Fig. 4) using the MBK clustering algorithm, a modification of the traditional k-means algorithm^119^. The regionalization process employed the meteorological and topographical variables listed in Table 4. These variables consist of both dynamic and invariant components, where the dynamic variables were used on a daily basis and the invariant variables retained constant values throughout the entire study period. Except for categorical variables such as precipitation type and vegetation type, as well as temporal and spatial coordinate indicators (year, month, latitude, and longitude), all variables were standardized using Z-scores. The traditional k-means algorithm is an unsupervised ML algorithm that assigns each data point to the nearest cluster and iteratively updates cluster centroids until convergence, thereby dividing the dataset into distinct, non-overlapping clusters^120,121^. However, this approach can be computationally intensive and inefficient due to the repeated use of all data points in each iteration. The MBK algorithm addresses this limitation by using a probabilistic approach during each iteration, significantly reducing computational costs for large datasets^122,123^.

To determine the optimal number of clusters, we employed three techniques: the elbow method^124^, the silhouette coefficient^125^, and the gap statistic^126^. The elbow method identifies the optimal cluster count by locating the point at which the reduction in the sum of squared errors (SSE) between points and their respective cluster centroids begins to level off. The silhouette coefficient measures the average distance between a data point and other points in the same cluster relative to the average distance to points in other clusters; higher silhouette coefficients indicate better cluster quality. The gap statistic compares the total intra-cluster variation for different numbers of clusters with the expected variation under an appropriate reference null distribution (i.e., a distribution with no clustering), identifying the point at which the gap statistic reaches its maximum. The number of reference null distributions used in calculating the gap statistics is determined by the user. In this study, we used 500 reference null distributions, as suggested by Iwasawa *et al*.^127^, which provided stable gap statistics.

The optimal number of clusters was determined using the silhouette coefficient, beginning from the point at which the SSE is minimized and the gap statistic is maximized. Based on the intersection of the elbow and gap statistics, the minimum number of clusters was identified as 10, while the silhouette coefficient indicated an optimal number of 14. Therefore, the 11,687 grid points were partitioned into 14 clusters (Fig. 4). The resulting regionalization exhibits spatial patterns consistent with the distribution of *in-situ* measurements, as geographical characteristics (e.g., latitude and longitude) were included, naturally aligning the clusters with the measurement network. However, the clustering results do not merely replicate the network distribution; they also integrate sites from multiple networks when their meteorological and geographical characteristics are similar. In North America, the region was broadly divided into five clusters, reflecting distinctive features in areas such as Alaska and the Rocky Mountains. Eurasia was divided into three major clusters: Europe, the Far East, and inland Central Siberia. China was divided into six clusters, representing the largest number, which reflects both the substantial variability in meteorological and geographical characteristics^128^ and the highest density of grid points. In addition, Cluster 14 was classified as regions where snowfall is rarely observed, including Africa, Florida, the Amazon, and Southeast Asia.

### ML algorithm

#### General model design

We employed the RF algorithm to generate a global SWE product, as illustrated in the schematic in Fig. 1. Random Forest (RF) is an ensemble learning algorithm based on decision trees, widely used for both classification and regression tasks due to its high accuracy and computational efficiency^129^. RF operates on the principle of bootstrap aggregating (bagging), in which bootstrapped datasets are generated by randomly selecting samples with replacement for each decision tree. This approach reduces overfitting by averaging predictions across multiple decision trees, making the model more robust to variations in training data, noise, and outliers, thereby enhancing predictive performance and overall stability^130,131^. Each decision tree in an RF model is trained on a bootstrap sample, leaving some data points unused in the training process (referred to as out-of-bag, or OOB, samples). A key advantage of this method is that OOB samples can be used to evaluate model performance without requiring a separate validation set. In this study, we developed the RF model using the scikit-learn library in Python^132^ and tuned its hyperparameters using GridSearchCV.

As described in Fig. 1 and in the Regionalization section, we applied the MBK algorithm to group the 11,687 grid points into 14 clusters. Regionalization enhances the training accuracy and efficiency of ML models by incorporating diverse *in-situ* SWE data from a wide range of global sites. Two types of RF models were employed to generate the SWEML product. Hyperparameters for both models were tuned using a grid search, selecting the parameter sets at the point where performance metrics (i.e., the best score) no longer showed substantial improvement. First, we built a RF regression (RFR) model for each cluster to learn the relationship between rescaled *in-situ* SWE measurements (adjusted using ERA5-Land SWE; see the Data screening and rescaling section) and meteorological forcing variables and terrain features. The number of trees (n_estimators) was set to 400, the random_state parameter was fixed at 42, and the number of variables considered at each split (max_features) was set to “sqrt” (i.e., the square root of the total number of input variables). All other parameters were kept at their default values in scikit-learn. Each model was trained using 70% of the data and tested on the remaining 30%. Consequently, the 14 independent RFR models capture regional variability in meteorological and geographical characteristics and directly estimate SWE for each grid cell using the relevant input data. Second, we built an RF classification (RFC) model to learn the relationship between cluster assignment and meteorological forcing variables and terrain features. For the RFC model, the n_estimators and class_weight parameters were set to 2000 and “balanced,” respectively, with random_state fixed at 42. This configuration assigns a greater penalty to misclassifications of minority classes compared to majority classes, improving performance on imbalanced datasets. The RFC model was trained using 80% of the data and tested on the remaining 20%. Using this model, each grid cell in the study domain was assigned to one of the 14 clusters (Fig. S3). SWE was then estimated for each grid cell using the RFR model corresponding to its assigned cluster, thereby constructing the final SWEML product.

#### Model evaluation

To ensure a robust assessment of model performance, we employed three evaluation approaches. Two types of cross-validation (CV) were applied to the RFR models for SWE estimation, and one type of CV was applied to the RFC model for cluster assignment. For the RFR models, we first used leave-one-site-out CV (LOSOCV), a modified form of Jackknife resampling. In this approach, all data from a single grid point are excluded during training, and model performance is evaluated using the excluded point. This method is particularly useful for assessing the robustness of SWE estimates at independent grid points without *in-situ* measurements. LOSOCV was applied to each RFR model and repeated for all grid points within each cluster. We then employed a 10-fold CV, which partitions the dataset into 10 equally sized folds, sequentially training the model on nine folds and validating it on the remaining fold. This approach evaluates the generalization ability of the model to independent samples and helps identify potential issues such as overfitting. The predictive performance of each RFR model was quantified using three statistical metrics: the coefficient of determination (*R*^2^), the Pearson correlation coefficient (*R*), and the standard deviation of residuals.

For the RFC models, we used stratified 10-fold CV, which preserves the original class distribution within each fold. Training and evaluating the model across multiple subsets reduces uncertainty in estimating generalization performance that may arise from reliance on a single dataset. This method provides a more reliable assessment of the classification model’s performance. Predictive performance was evaluated using overall accuracy, weighted precision, weighted recall, weighted F1-score, and the area under the receiver operating characteristic curve (ROC-AUC).

#### Evaluation of SWEML

Each SWE product exhibits differences in spatial coverage and spatiotemporal resolution. Accordingly, we employed four approaches to evaluate the performance of SWEML in this study. First, we assessed accuracy by comparing SWEML and gridded reference datasets against raw *in-situ* measurements that were not rescaled. To ensure consistency in spatiotemporal coverage, we used only grids available across all datasets. Validation was performed based on the spatiotemporal extent of seven reference datasets, focusing on grids above 35°N and the period from 2013 to 2017. Only observed dates corresponding to the AMSR-E/AMSR2 rotation cycle were included. This evaluation used 1,563,254 data points (SWE > 0 mm) from 5,630 sites between 2013 and 2017. For spatial distribution analysis among gridded SWE datasets, all values, including zero SWE, were considered. Next, we evaluated the accuracy of all gridded SWE datasets using GAMMA over North America, following the approach of Mortimer *et al*.^133^. For GAMMA geolocation, the midpoint of each flight line was used, and data were matched to the native grid of each SWE dataset and averaged accordingly. To reduce oversampling in regions with spatially dense networks, all product-reference pairs were averaged within sequential 200 km windows, starting with the Alaska GAMMA flight midpoint, AK101. Since GAMMA can detect SWE values up to 1000 mm, performance metrics were calculated using non-zero SWE values ≤ 1000 mm, including only cases where both GAMMA and the corresponding SWE dataset met this threshold. This assessment tests how well each gridded product captures the spatial characteristics of SWE at its native resolution. Lastly, we evaluated SWEML over the Andes, a region without *in-situ* measurements used in ML training, using the high-resolution ADSWE dataset. This evaluation included SWEML as well as the GLDAS, AMSR-E/AMSR2, and MERRA-2 datasets, providing insight into the model’s performance in areas lacking training data.

Temporal characteristics were then evaluated using the annual peak SWE, defined as the maximum SWE within each snow water year. Peak SWE was calculated as the maximum daily mean SWE across all grid cells within the study area, excluding zero values. Peak SWE, typically observed at the end of the accumulation season, serves as a key indicator of both the final state of snow accumulation and the initial condition for the subsequent ablation season. This metric is critical for characterizing temporal snow dynamics and enables consistent intercomparison across datasets^134^. Additionally, we quantified the accuracy of SWEML by comparing it with *in-situ* measurements, ADSWE, and GAMMA using multiple metrics: root mean square error (RMSE), mean absolute error (MAE), Pearson correlation coefficient (R), bias, and Spearman correlation coefficient (corr). The equations for these accuracy metrics are described as follows:

2 $${RMSE}=\sqrt{\frac{\mathop{\sum }\limits_{i=0}^{n}{\left({r}_{i}-{m}_{i}\right)}^{2}}{n}},$$

3 $${MAE}=\frac{1}{n}\mathop{\sum }\limits_{i=1}^{n}\left|{r}_{i}-{m}_{i}\right|,$$

4 $${\rm{R}}=\frac{\sum \left({r}_{i}-\bar{r}\right)\left({m}_{i}-\bar{m}\right)}{\sqrt{\sum {\left({r}_{i}-\bar{r}\right)}^{2}\sum {\left({m}_{i}-\bar{m}\right)}^{2}}},$$

5 $${Bias}={r}_{i}-{m}_{i},$$

6 $${corr}=1-\frac{6{\sum }_{i=1}^{n}{\left({rank}\left({r}_{i}\right)-{rank}\left({m}_{i}\right)\right)}^{2}}{n({n}^{2}-1)},$$

where $n$ is the number of samples, ${r}_{i}$ is the SWE value of each dataset, ${m}_{i}$ is the corresponding evaluation value (*in-situ* measurement or reference dataset). Here, $\bar{r}$ and $\bar{m}$ represent the mean values of the SWE dataset and evaluation data, respectively, which are used in the calculation of R. For corr, the ranks of ${r}_{i}$ and ${m}_{i}$ are used instead of their original values.

## Data Records

The SWEML product can be freely downloaded from Zenodo (10.5281/zenodo.16822772)^135^. This dataset provides data with a daily temporal resolution and 0.25° (approximately 25 km) spatial resolution. Furthermore, it covers global latitudes from 90°S to 90°N and longitudes from 180°W to 180°E, excluding Antarctica. The dataset is provided in NetCDF format, organized by year. Each year contains daily SWE data, including leap days in leap years. The coordinate system is WGS84 (“EPSG:4326”).

## Technical Validation

### Evaluating the performance of RF algorithms

**Fig. 5:**
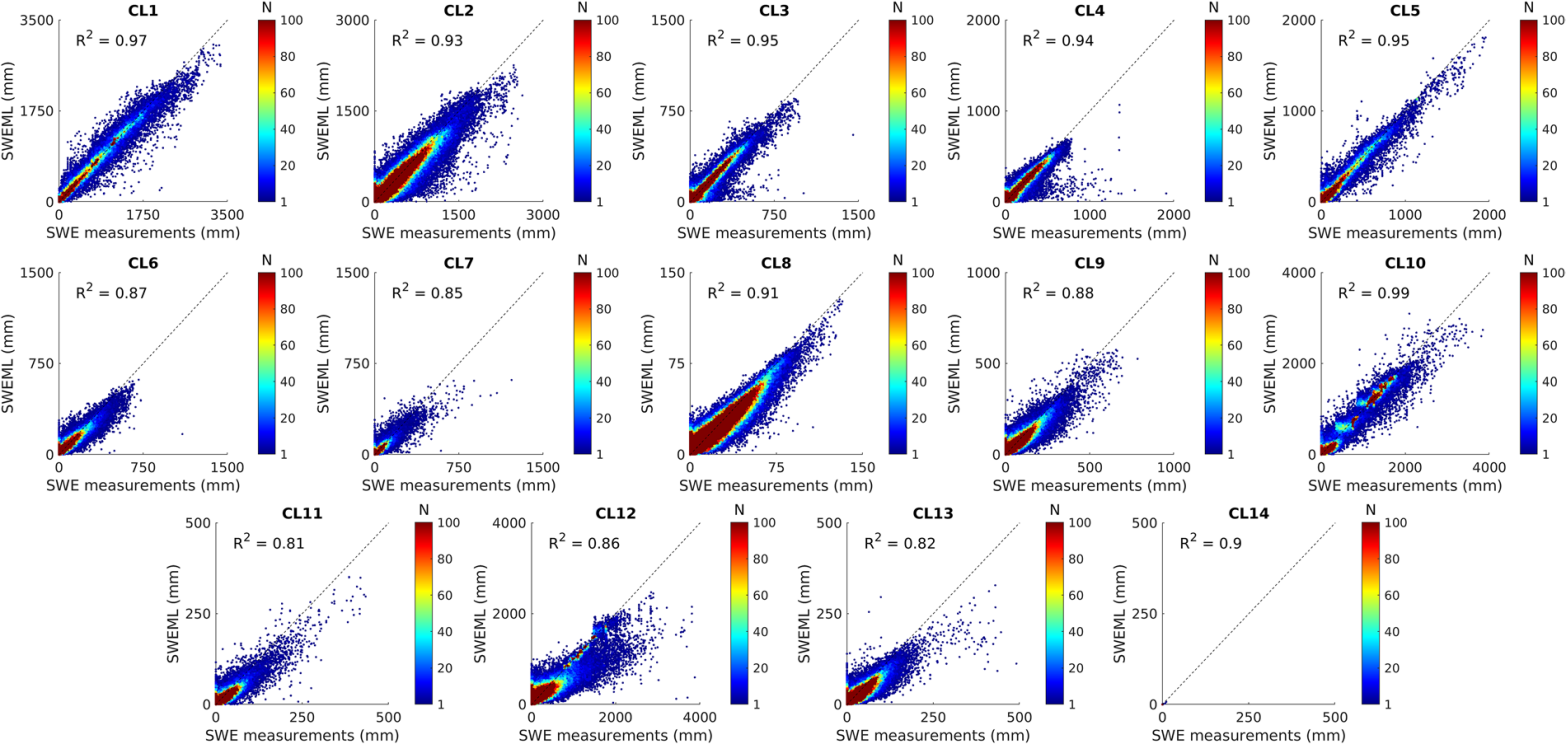
Scatterplots comparing SWEML estimates with corresponding *in-situ* measurements across all grid cells within each cluster.

**Fig. 6:**
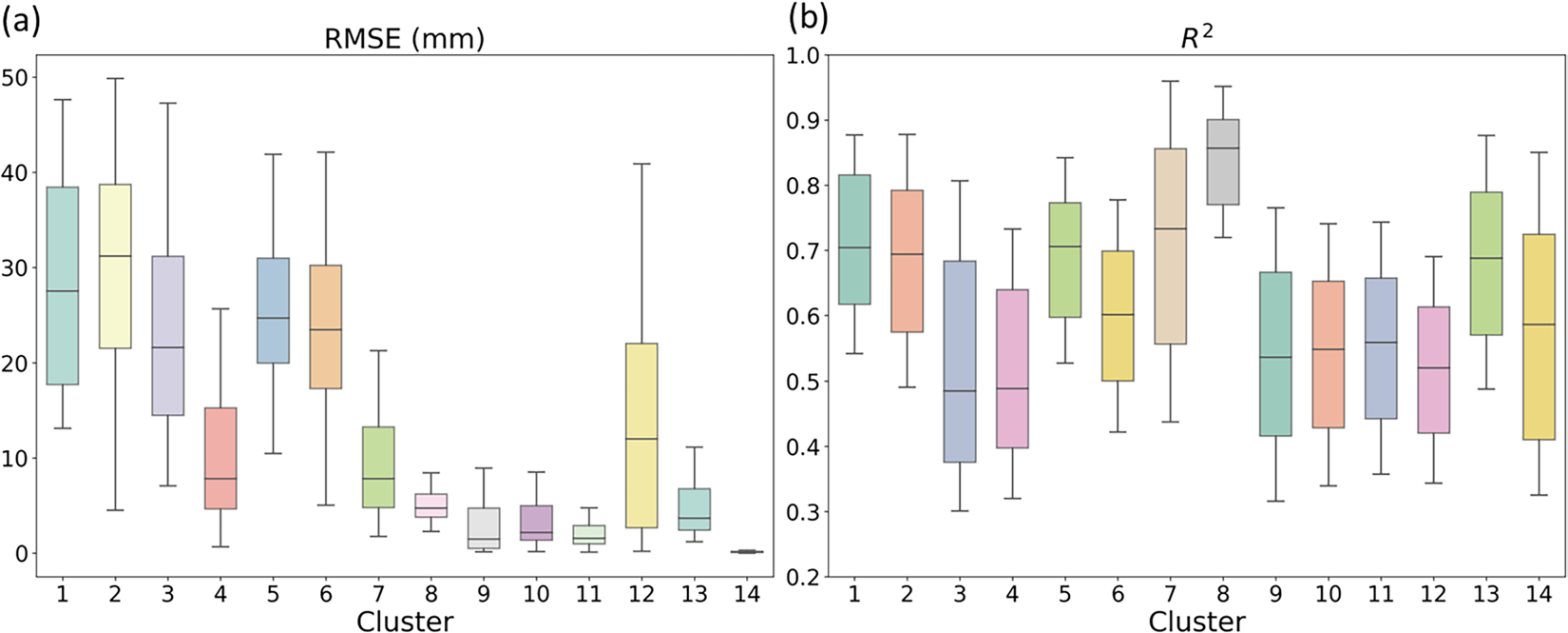
Box plots of the (**a**) RMSE (mm) and (**b**) R^2^ by cluster from the LOSOCV evaluation of the RFR model across the 14 clusters. Boxes indicate the interquartile range (25th–75th percentiles), the horizontal line marks the median, and whiskers represent the minimum and maximum values.

The predictive accuracy of the RFR model was evaluated across the 14 clusters (Figs. 5, 6). The R^2^ values between SWEML and *in-situ* SWE measurements ranged from 0.81 to 0.99, indicating strong model reliability and high predictive skill (Fig. 5). Clusters 1, 2, 3, 5, and 10, corresponding to snow-dominated regions such as Alaska, the Rocky Mountains, the Alps, Scandinavia, and the Himalayas, exhibited R^2^ values above 0.93, demonstrating that the RFR models accurately captured the high SWE variability in these areas. These results were consistent with the 10-fold CV (Table S1), which showed low standard deviations across folds, suggesting that the models maintained both high accuracy and stable performance under diverse snow conditions.

We next evaluated model performance under spatially independent conditions by employing LOSOCV. As expected, the predictive skill decreased moderately compared to the standard evaluation, reflecting the challenge of predicting values at ungauged locations. Nevertheless, the models retained reasonable performance, with the R^2^ generally ranging between 0.524 and 0.806 and the RMSE varying regionally but remaining within acceptable ranges (Table S2). The relatively narrow interquartile ranges for the R^2^ and RMSE across many of the clusters indicated consistent prediction error, highlighting the stability of the model even under spatially independent conditions. Notably, clusters 3, 4, 9, 10, 11, 12, and 14 exhibited relatively low R^2^ alongside low RMSE. For cluster 14, this pattern arises because the model predicts very low SWE values in the LOSOCV evaluation, and therefore, even minor errors contribute substantially to the total variance. In the other clusters, the heterogeneity in SWE values and temporal dynamics among stations increases residual variance for some test stations, lowering R^2^ without substantially affecting RMSE. Overall, these results demonstrate that the RFR models provide robust SWE estimates for ungauged locations, supporting their applicability in data-sparse or observationally limited regions.

The RFC model, used to assign each grid cell to one of the 14 clusters, was evaluated using stratified 10-fold CV. The overall accuracy was 0.895, with nearly identical values across all folds and a very low standard deviation of 0.0006, indicating highly stable model performance (Table S3). Precision and recall were 0.919 and 0.895, respectively, suggesting balanced predictions without bias toward any specific class. The F1-score of 0.898 demonstrates a stable balance between precision and recall, ensuring consistent performance across clusters. The ROC-AUC reached 0.992, indicating excellent class discrimination and strong predictive capability even with imbalanced datasets. All metrics had standard deviations below 0.0001, confirming that the model’s predictions remained consistent regardless of data partitioning.

### Performance of SWEML

**Fig. 7:**
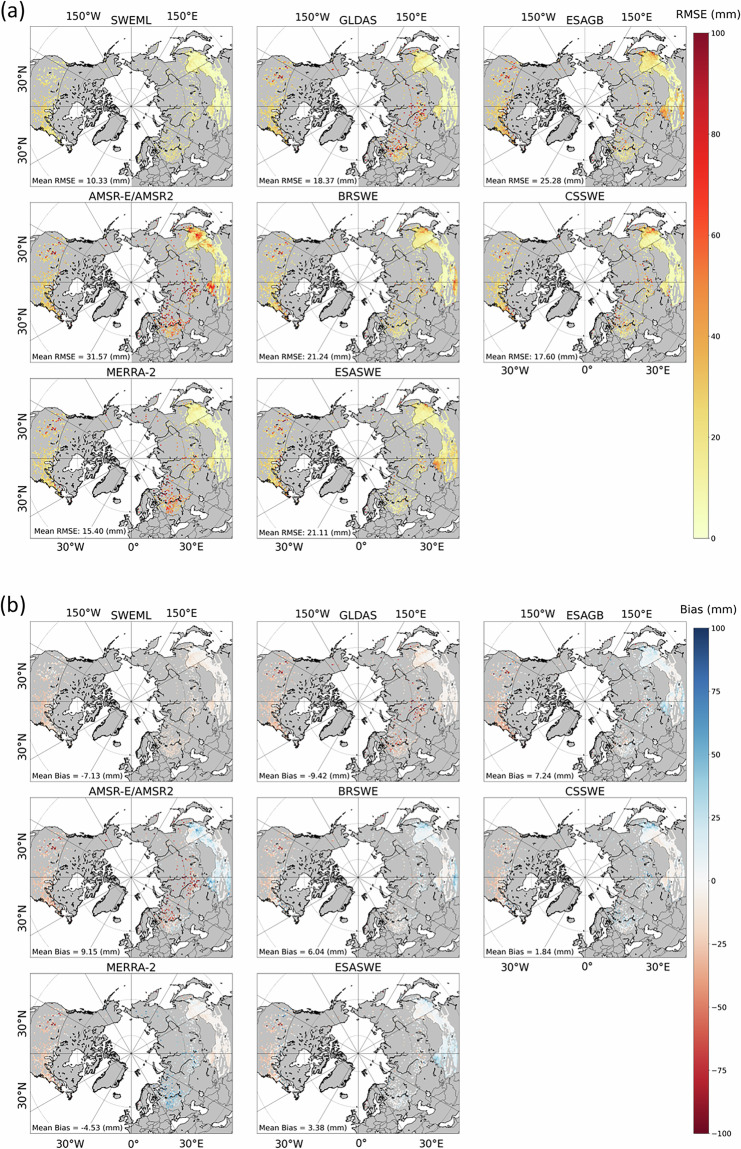
Comparison of SWEML with the reference SWE products (GLDAS, ESAGB, AMSR-E/AMSR2, BRSWE, CSSWE, MERRA-2, and ESASWE): (**a**) RMSE (mm) and (**b**) bias (mm).

**Fig. 8:**
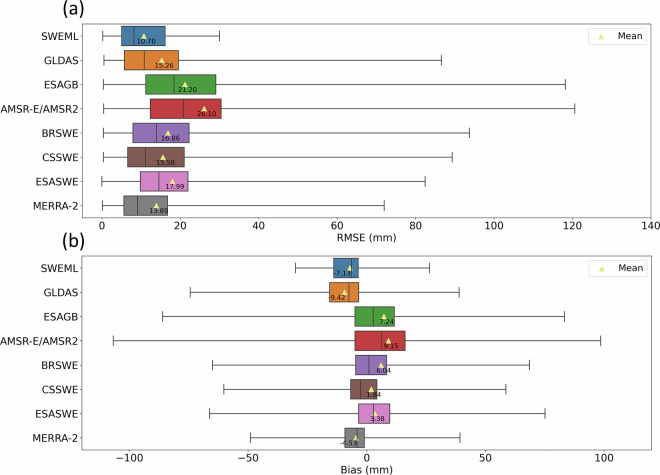
Spear box plots of the (**a**) RMSE (mm) and (**b**) bias of each SWE product at all *in-situ* measurement sites. The left and right edges of each box represent the 25^th^ and 75^th^ percentiles, respectively. The solid vertical line within each box indicates the median value, while the whiskers denote the minimum and maximum values.

We next compared the accuracy of SWEML with seven reference datasets (GLDAS, ESAGB, AMSR-E/AMSR2, BRSWE, CSSWE, MERRA-2, and ESASWE) for the 2013–2017 period using raw, non-rescaled *in-situ* measurements (Fig. 7). SWEML exhibited a spatial mean RMSE of 10.33 mm, ranging from 0.23 mm to 32.43 mm, which is lower than the RMSEs of all other products (GLDAS: 18.37 mm; ESAGB: 25.28 mm; AMSR-E/AMSR2: 31.57 mm; BRSWE: 21.24 mm; CSSWE: 17.60 mm; MERRA-2: 15.40 mm; ESASWE: 21.11 mm) (Figs. 7a, 8a). The spatial distribution of RMSEs indicates that SWEML is substantially more accurate in snow-dominant regions such as Alaska, the Rocky Mountains, the Scandinavian Peninsula, China, and the Far East. For instance, in the Rocky Mountains, SWEML achieved an RMSE of 16.51 mm, representing a 68.35% reduction compared to CSSWE, the dataset with the second-lowest RMSE in this region. Among the reanalysis datasets, accuracy varied regionally. Although GLDAS had a lower spatial mean RMSE than ESAGB, BRSWE, and AMSR-E/AMSR2, it exhibited high RMSEs in Alaska (70.31 mm) and the Rocky Mountains (124.13 mm). ESASWE, which shares a similar algorithm with ESAGB, improved upon ESAGB’s accuracy, consistent with Mortimer *et al*.^69^, who mentioned that ESASWE represents an improvement over ESAGB, which is based on a similar algorithm to Snow CCI + version 1. However, it still shows high RMSEs in the Scandinavian Peninsula. MERRA-2 performed reasonably well, with a spatial mean RMSE of 15.40 mm and relatively low errors across all regions. While our approach differs from that of Mudryk *et al*.^76^, our results are consistent with their finding that the performance rank of MERRA-2 is higher than that of ESAGB, ESASWE, and GLDAS. For the physically based models, CSSWE and BRSWE exhibited relatively stable performance. CSSWE showed reasonable accuracy with consistently low RMSEs across all regions. Notably, in areas such as the Rocky Mountains and the Scandinavian Peninsula, where other products exhibited high RMSEs, CSSWE maintained reasonable accuracy with lower errors, consistent with previous findings that rank it among the top-performing SWE products. In contrast, AMSR-E/AMSR2 exhibited the highest spatial mean RMSE among all eight datasets, particularly in Alaska, the Rocky Mountains, the Scandinavian Peninsula, the Russian Far East, and China, with RMSEs of 70.84 mm, 116.74 mm, 255.41 mm, 28.61 mm, and 29.45 mm, respectively. These results indicate that SWEML most accurately captures SWE variability as observed in *in-situ* measurements.

In terms of bias (Fig. 7b), SWEML, GLDAS, and MERRA-2 exhibited negative spatial mean biases of −7.13 mm, −9.42 mm, and −4.53 mm, respectively, whereas the other SWE datasets showed positive spatial mean biases. Regional variations in bias were generally large for all datasets except GLDAS and SWEML. SWEML slightly underestimated SWE in most regions, with negative biases, except in Alaska and the Scandinavian Peninsula, where it slightly overestimated SWE with positive biases. In contrast, GLDAS consistently underestimated SWE across most regions (Table 5), with particularly large negative biases in the Rocky Mountains and the Scandinavian Peninsula. MERRA-2 showed a similar pattern of large negative biases but captured positive biases in European Russia and a relatively high positive bias of 30.57 mm in Alaska.

**Table 5:** Statistical metrics of each SWE product by region.

Region	Statistical metric	SWE Product							
		SWEML	GLDAS	ESAGB	AMSR-E/AMSR2	BRSWE	CSSWE	ESASWE	MERRA-2
Alaska	RMSE (mm)	16.72	70.31	45.76	70.84	45.20	38.51	35.71	49.84
	NMAE (%)	0.16	0.62	0.41	0.66	0.39	0.30	0.30	0.43
	R	0.95	0.23	0.64	0.56	0.67	0.80	0.76	0.84
	Bias (mm)	2.94	−45.55	−10.24	−57.50	−25.73	9.67	−8.65	30.57
Rocky Mountains	RMSE (mm)	16.51	124.13	103.05	116.74	85.99	52.17	95.02	98.92
	NMAE (%)	0.15	0.86	0.70	0.84	0.60	0.36	0.64	0.68
	R	0.99	0.47	0.6	0.63	0.80	0.90	0.66	0.71
	Bias (mm)	−4.26	−79.87	−60.42	−77.63	−53.50	−26.56	−51.86	−62.70
Scandinavia	RMSE (mm)	18.39	115.22	198.91	255.41	174.37	68.26	255.91	105.16
	NMAE (%)	0.08	0.41	0.67	0.88	0.60	0.22	0.83	0.36
	R	0.99	0.92	0.82	0.66	0.87	0.95	0.34	0.91
	Bias (mm)	3.42	−77.65	−128.68	−171.88	−117.00	−4.61	−163.32	−58.22
Russian Far East	RMSE (mm)	10.65	14.96	21.43	28.61	18.88	20.07	14.40	11.84
	NMAE (%)	0.42	0.54	0.78	0.83	0.66	0.63	0.49	0.41
	R	0.87	0.61	0.57	0.32	0.50	0.61	0.57	0.68
	Bias (mm)	−7.59	−9.3	8.38	7.83	8.32	7.63	1.08	−2.18
China	RMSE (mm)	10.10	13.27	23.27	29.45	19.41	16.70	18.15	11.22
	NMAE (%)	0.52	0.63	1.06	1.19	0.87	0.71	0.78	0.53
	R	0.84	0.64	0.53	0.36	0.47	0.64	0.43	0.70
	Bias	−6.97	−8.31	8.15	11.94	6.9	1.59	4.51	−48.1

The physically based models BRSWE and CSSWE exhibited similar bias patterns and spatial distributions across most regions, except for Alaska. CSSWE showed the lowest bias value, with a positive spatial mean bias at 1.84 mm and generally lower biases than other datasets across snow-dominated regions. In contrast, BRSWE exhibited higher biases than CSSWE. Both models showed positive biases, slightly overestimating SWE in China and the Russian Far East. In Alaska, however, BRSWE and CSSWE exhibited opposite bias directions, with one showing negative and the other positive deviations. ESAGB and ESASWE displayed similar spatial bias distributions, consistent with their RMSE patterns, with ESASWE showing reduced biases and overall improvement compared to ESAGB across all regions. ESAGB tended to underestimate SWE with large negative biases, particularly in high-elevation regions of central Eurasia and the Kamchatka Peninsula, resulting in slightly higher RMSEs, a pattern also noted by Luojus *et al*.^22^. In ESASWE, parts of these high-elevation regions showed reduced RMSEs and lower biases, indicating improvements (Fig. 7). Among all datasets, AMSR-E/AMSR2 exhibited the highest RMSE and the largest spatial mean bias of 9.15 mm, with substantial biases across most regions.

SWEML exhibited lower biases than the reference datasets across various regions, with spatial mean biases of 2.94 mm, −4.26 mm, 3.42 mm, −7.59 mm, and −6.97 mm, respectively (Table 5). Notably, SWEML improved the absolute bias by 5.71 mm, 22.3 mm, and 1.19 mm in Alaska, the Rocky Mountains, and the Scandinavian Peninsula, respectively, compared with the dataset showing the lowest bias in each region. In the Russian Far East and China, SWEML showed higher biases than ESASWE and CSSWE, respectively. However, despite these higher biases, SWEML achieved greater overall accuracy in these regions, with RMSEs of 10.65 mm and 10.10 mm and R of 0.87 and 0.84, respectively.

The ranges of RMSE and bias for each SWE product are shown in Fig. 8. SWEML exhibited a small interquartile range (IQR) of RMSE and the lowest maximum RMSE of 32.43 mm, indicating robust performance (Fig. 8a). In contrast, the reference datasets presented relatively wider RMSE IQRs, suggesting they captured the variability in *in-situ* measurements less effectively. The maximum RMSEs of GLDAS, ESAGB, AMSR-E/AMSR2, BRSWE, CSSWE, ESASWE, and MERRA-2 were 86.57 mm, 118.15 mm, 120.60 mm, 93.72 mm, 89.33 mm, 82.42 mm, and 71.98 mm, respectively. The bias of SWEML ranged from −31.27 mm to 26.46 mm, which was smaller than that of MERRA-2 (−49.01 mm to 39.24 mm) and much smaller than CSSWE, which varied from −60.17 mm to 58.61 mm. The low mean bias of CSSWE resulted from the offset between its large positive and negative biases, whereas SWEML demonstrated both a lower mean bias and smaller variability, reflecting greater accuracy. AMSR-E/AMSR2 exhibited the widest bias range, with −106.80 mm and 98.50 mm, respectively.

### Elevational and temporal characteristics

**Fig. 9:**
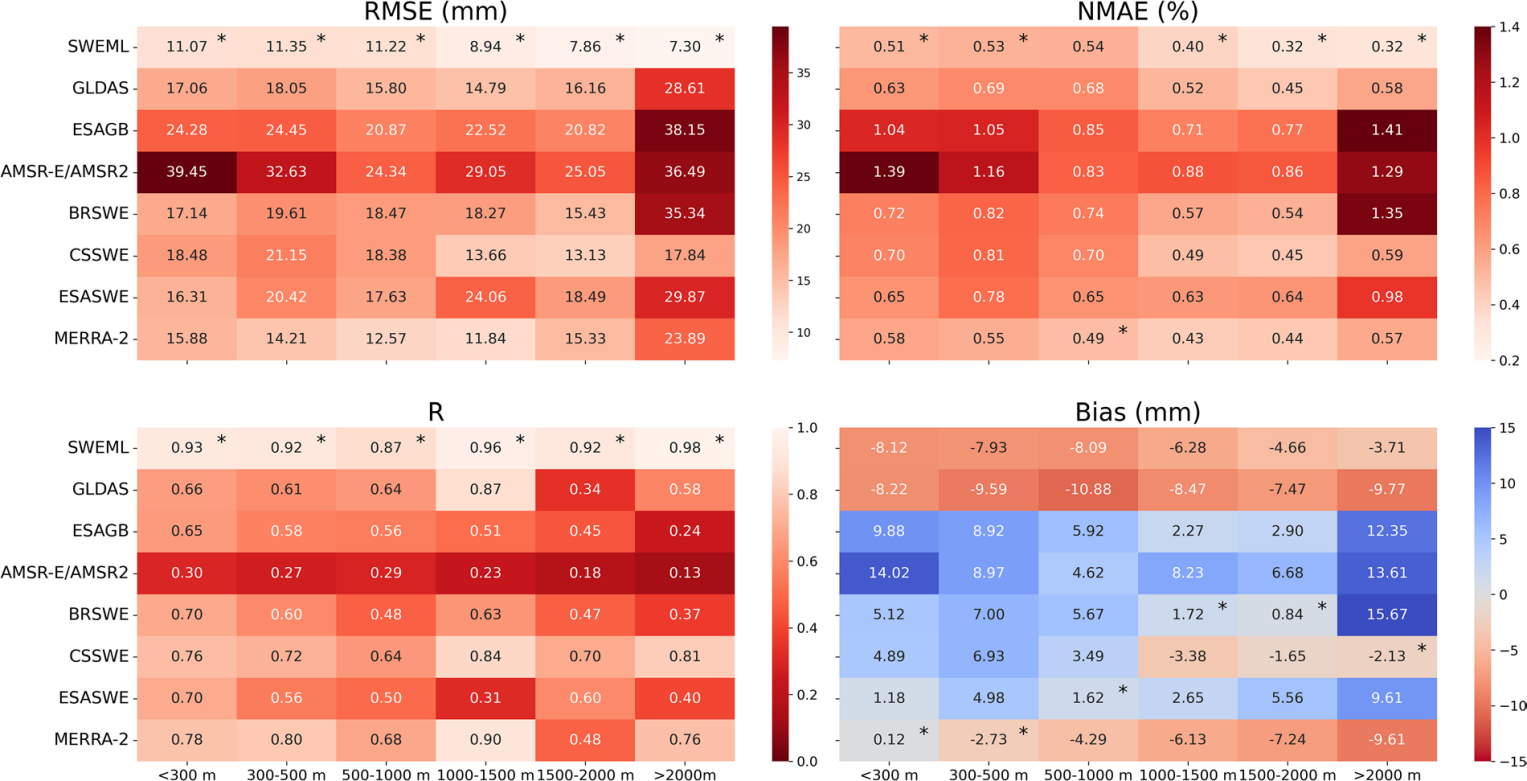
Comparison of SWEML with the reference products (GLDAS, ESAGB, AMSR-E/AMSR2, BRSWE, CSSWE, ESASWE, and MERRA-2) for different elevation ranges. Values marked with an asterisk (*) indicate the best-performing dataset for each elevation range.

The performance of SWEML was compared with seven reference datasets (ESAGB, GLDAS, AMSR-E/AMSR2, BRSWE, CSSWE, ESASWE, and MERRA-2) across different elevation ranges (Fig. 9). Although many raw *in-situ* measurements above 2000 m were available (Fig. 2b), a large number of these were excluded when harmonizing them with the reference datasets due to data availability. For example, ESASWE and ESAGB do not provide SWE values over grid cells corresponding to high-elevation regions. The analysis was therefore conducted using six elevation classes.

SWEML outperformed all reference datasets across elevations in terms of RMSE, normalized MAE (NMAE), and *R*. Notably, SWEML showed the best performance above 2000 m, where snow accumulation is highest, with an RMSE, NMAE, and *R* of 7.30 mm, 0.32%, and 0.98, respectively. In contrast, the other reference datasets had weaker performance at elevations above 2000 m. Specifically, the AMSR-E/AMSR2 dataset demonstrated the lowest accuracy at elevations above 2000 m, with an RMSE, NMAE, and R of 36.49 mm, 1.29%, and 0.13, respectively. This dataset also performed poorly at elevations below 300 m (RMSE = 39.45 mm, NMAE = 1.39%, *R* = 0.30, bias = 14.02 mm). CSSWE performed slightly worse than SWEML in terms of RMSE, NMAE, and *R*, but had lower bias (−2.13 mm). BRSWE showed a high positive bias above 2000 m (15.67 mm) but lower bias below 2000 m, overall demonstrating slightly smaller biases than SWEML.

**Fig. 10:**
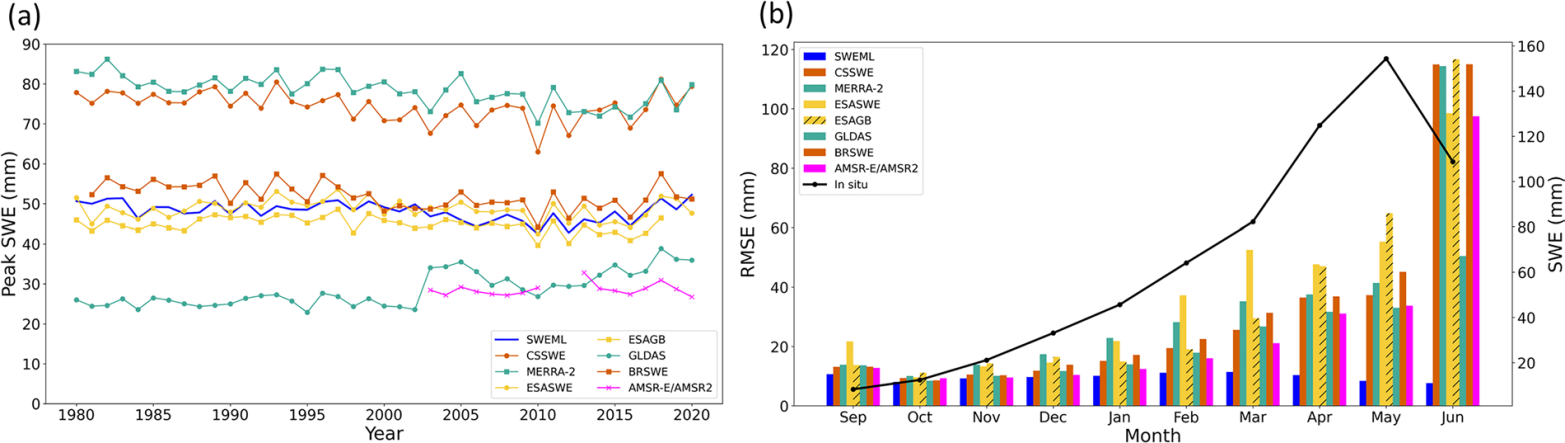
(**a**) Time series of peak SWE from SWEML and reference products. (**b**) Monthly mean RMSE (mm) of the SWE products compared with *in-situ* measurements (left y-axis). The black line represents the monthly mean SWE (mm) from *in-situ* measurements (right y-axis).

To assess temporal performance, we compared the peak SWE time series across datasets (Fig. 10a). MERRA-2 and CSSWE showed relatively high peak values with similar trends, while SWEML, ESAGB, ESASWE, and BRSWE exhibited closely matching trends and peak SWE values. GLDAS showed increasing peak SWE after 2003, corresponding to the transition from GLDAS v2.0 to v2.2, although annual peaks remained relatively low. We further evaluated seasonal representation by comparing *in-situ* measurements with monthly averages of each dataset (Fig. 10b). Across the snow water year (September–June), *in-situ* SWE increased until May before declining. The reference datasets generally showed low RMSEs in winter, but differences increased from March onward, peaking in June. SWEML consistently had the lowest RMSEs across all months, indicating reliable seasonal performance. These results demonstrate that, despite rescaling *in-situ* measurements were standardized to the ERA5-Land scale in terms of mean and variance, their inherent temporal variability was effectively preserved. Consequently, these results highlight the model’s ability to effectively capture seasonal features of *in-situ* measurements.

### Spatial comparison among the SWE datasets

**Fig. 11:**
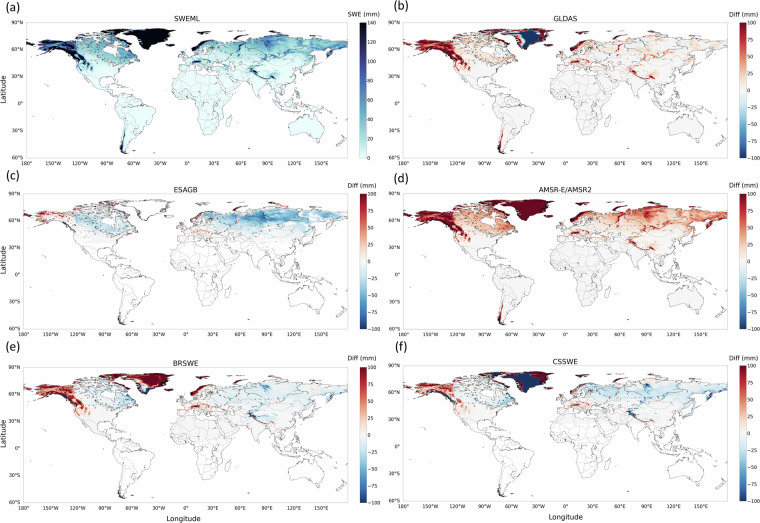
(**a**) Annual mean SWE derived from SWEML. Differences between SWEML and the (**b**) GLDAS, (**c**) ESAGB, (**d**) AMSR-E/AMSR2, (**e**) BRSWE, and (**f**) CSSWE SWE datasets.

Figure 11 and Fig. S4 illustrate the spatial distribution of SWEML in comparison with seven reference datasets. Overall, SWEML showed high SWE values across major snow-dominated regions, including the Rocky Mountains, the Alps, the Himalayas, the Urals, the Andes, Alaska, and the Scandinavian Peninsula (Fig. 11a). When compared with the five reference datasets of the same spatial resolution (GLDAS, ESAGB, AMSR-E/AMSR2, BRSWE, and CSSWE), SWEML exhibited broadly consistent spatial patterns (Fig. 11b–f). However, GLDAS and AMSR-E/AMSR2 generally produced lower SWE estimates than SWEML in most snow-dominated regions, including the Rocky Mountains, Alaska, the Scandinavian Peninsula, the Alps, the Himalayas, the Urals, and the Andes, as indicated by the large positive values in Fig. 11b and 11d. These low SWE estimates for GLDAS have also been reported by Shao *et al*.^25^. Both GLDAS and AMSR-E/AMSR2 had particularly low SWE in northeastern Canada, Siberia, and the Kamchatka Peninsula compared to SWEML (Fig. 11b,d). The ESAGB dataset, which covers parts of the Northern Hemisphere above 35° latitude but contains many gaps (Fig. 11c), exhibited lower SWE values in Europe and Alaska, but higher values across northeastern Canada and much of the Eurasian continent relative to SWEML. The model-based products BRSWE and CSSWE, which cover parts of the Northern Hemisphere above 10° and 25° latitude, respectively, showed spatial differences similar to those of SWEML (Fig. 11e,f). Both datasets showed lower SWE in Alaska, the western United States, the Alps, the Scandinavian Peninsula, and the eastern Himalayas, while higher SWE values were observed in northeastern Canada, Siberia, the Tibetan Plateau, and the Kamchatka Peninsula compared with SWEML.

The spatial distributions of ESASWE and MERRA-2, which have different resolutions, show noticeable deviations from SWEML in certain regions (Fig. S4). ESASWE, which excludes some complex regions and therefore has more limited coverage, exhibited lower SWE values in Europe and Alaska but higher values across northeastern Canada and the Eurasian continent compared with SWEML (Fig. S4b). This pattern is similar to that of ESAGB, as ESASWE was developed from the ESAGB algorithm and can be regarded as its improved version. This is also consistent with earlier results, as ESASWE showed improvements over ESAGB in nearly all evaluation metrics (Figs. 7–9). In contrast, MERRA-2 produced lower SWE estimates in the Rocky Mountains, the Alps, the Himalayas, the Andes, and northern Alaska, but higher values in Siberia and the Kamchatka Peninsula (Fig. S4c).

### Regional comparison among the SWE datasets

**Fig. 12:**
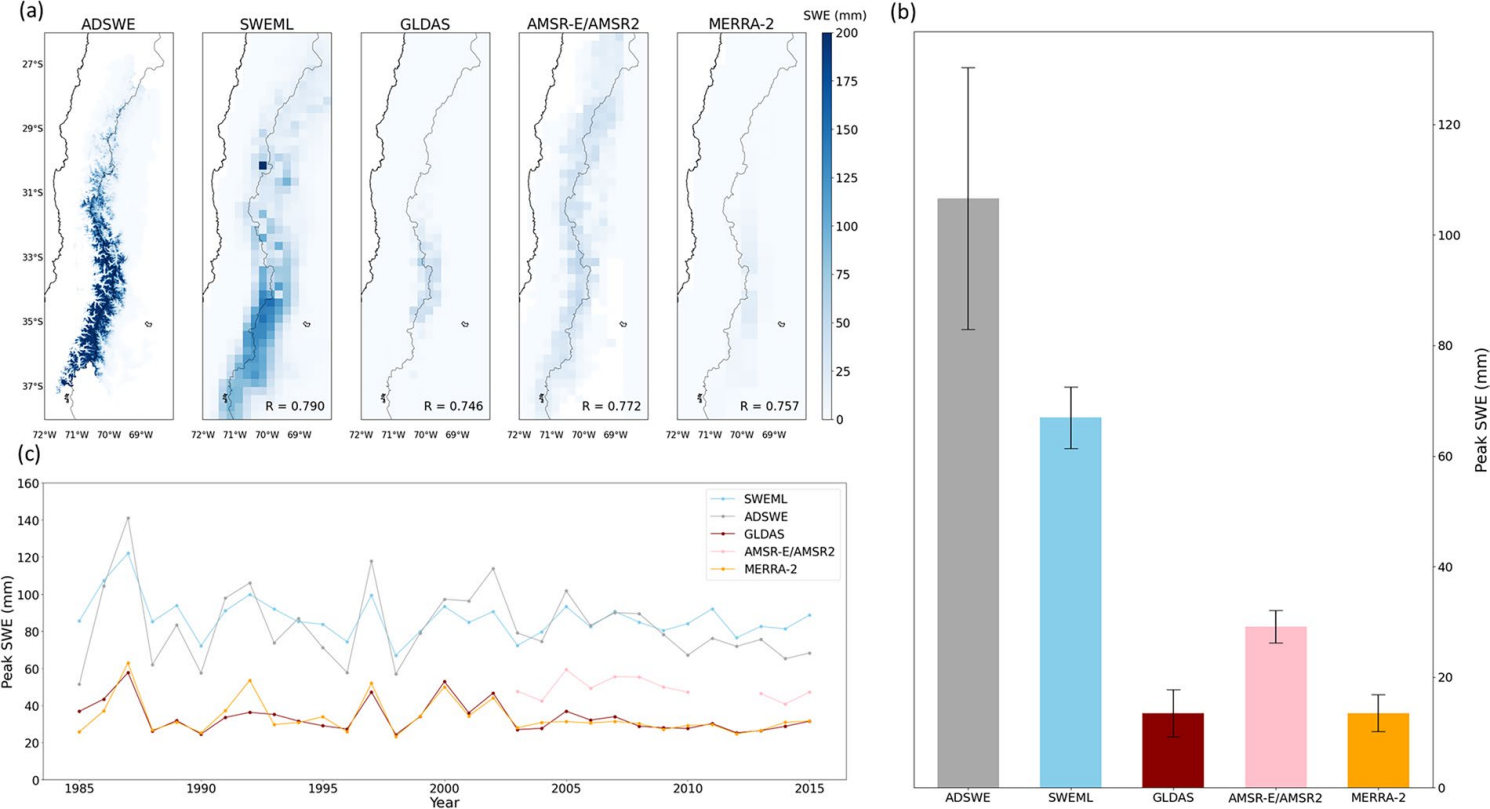
(**a**) Spatial distribution of SWE from each product. R indicates the correlation between the annual mean nonzero peak SWE across all grid cells for each product and ADSWE. (**b**) Mean peak SWE averaged over all grid cells, with solid lines showing the range across individual grid cells. (**c**) Time series of mean nonzero peak SWE from each product. The AMSR-E/AMSR2 data cover 2003–2020, except for 2011 and 2012, while the other datasets cover 1985–2020. Zero values were excluded from both the peak SWE time series and the spatial mean calculations. Panels (**b,c**) exclude glacier-covered areas based on the glacier mask.

We further evaluated regional performance by focusing on North America and the Andes, using the widely applied high-resolution GAMMA and ADSWE datasets as individual reference datasets. In the Andes, SWEML produced spatial patterns that were more consistent with ADSWE than with GLDAS, AMSR-E/AMSR2, or MERRA-2 (Fig. 12a). Notably, the correlation of annual mean peak SWE (excluding zeros) between SWEML and ADSWE was 0.79, the highest among all of the datasets. Although AMSR-E/AMSR2 also exhibited visually similar spatial patterns in this region, its SWE estimates were lower. The high-resolution ADSWE effectively captured complex topography, resulting in a higher peak SWE and a wider SWE range (Fig. 12b). By contrast, SWEML showed lower peak values and a narrower range than ADSWE but still achieved substantial improvements relative to the three reference datasets. Moreover, the time series of peak SWE was highly consistent with the temporal characteristics of ADSWE (Fig. 12c). Other reference datasets also showed similar temporal trends but consistently underestimated SWE, whereas SWEML provided more reasonable estimates.

**Fig. 13:**
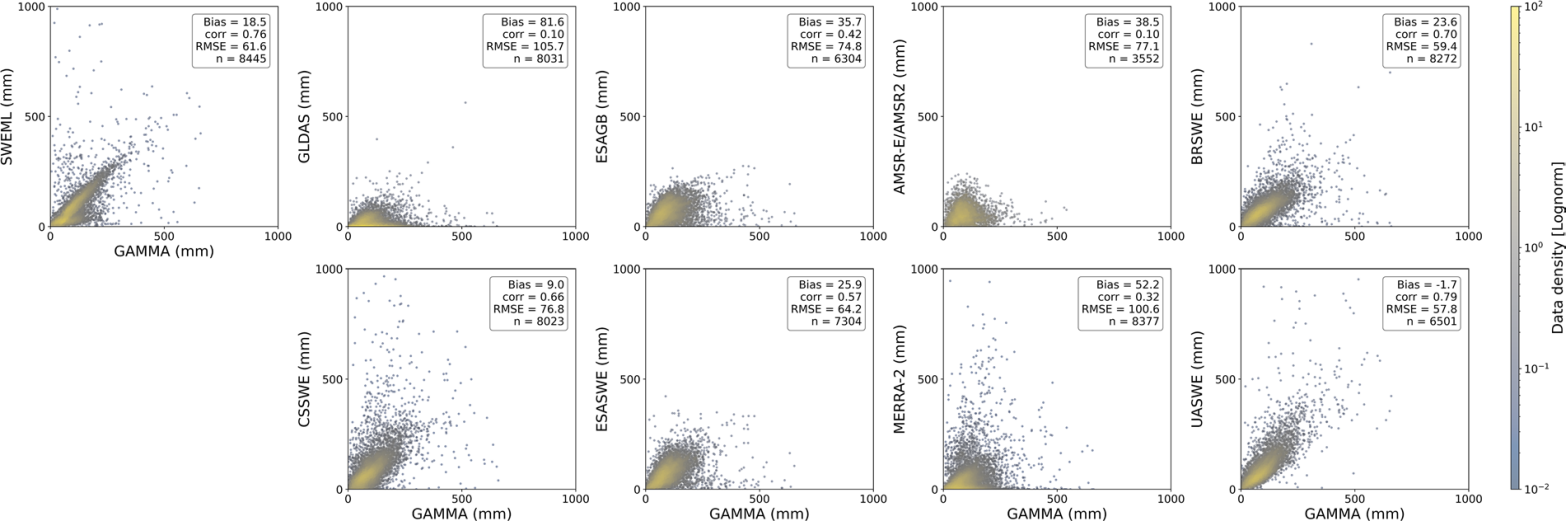
Scatterplots of product versus reference SWE density for measurements >0 mm and <1000 mm during the November–May. ESAGB and ESASWE exclude mountainous areas, and UASWE is limited to CONUS. See Table 2 for product names and corresponding time periods.

The relationships between GAMMA and all SWE datasets, including SWEML, are presented for North America in Fig. 13, showing clear differences in performance among the datasets. UASWE, with a bias of −1.7 mm and a corr of 0.79 with GAMMA, outperformed all products regardless of the reference dataset; however, its coverage was limited to CONUS, which restricted the validation statistics. The strong consistency of UASWE is in agreement with many previous studies^89,133^. When considering the broader North American region, including Alaska and Canada, the model-based BRSWE and CSSWE exhibited strong correlations, with values of 0.70 and 0.66, respectively. However, CSSWE exhibited a higher RMSE of 76.8 mm, while BRSWE had a higher bias of 23.6 mm. In contrast, AMSR-E/AMSR2, MERRA-2, and GLDAS exhibited the weakest performance, with a corr of 0.10, 0.32, and 0.10, respectively, indicating that their SWE estimates had a very weak statistical relationship with the observed SWE. The performance of ESAGB and ESASWE was moderate but, as reported by Mortimer *et al*.^133^, their estimates became saturated when the reference SWE exceeded approximately 250 mm. Similar to CSSWE and BRSWE, SWEML had the largest number of data pairs and a strong correlation with UASWE, with a corr of 0.76.

## Usage Notes

### Known limitations

Although SWEML is a globally gap-free SWE product in both space and time, several limitations should be considered prior to its use. SWEML has a spatial resolution of 0.25°, which may be insufficient to fully capture complex mountainous terrain and heterogeneous land cover in regions where SWE plays a critical role. Although the comparison with ADSWE demonstrates substantial improvement over GLDAS, AMSR-E/AMSR2, and MERRA-2, SWEML still tends to underestimate deep-snow conditions in complex mountainous regions, which remains a significant challenge in terms of the spatial resolution (Fig. 12b). In addition, our evaluation of model performance in mountainous areas relied exclusively on the elevations of sites where *in-situ* measurements were available. Therefore, evaluating the SWE prediction performance in complex mountainous terrain remains a significant challenge. Snow has been classified into approximately seven characteristic types based on land cover^136,137^. At the spatial scale, our analysis was robustly validated from the continental to the regional scale using ten reference datasets, including the high resolution ADSWE and the spatially representative GAMMA. However, further evaluation remains challenging, particularly in assessing model performance with respect to SWE characteristics classified by land cover.

In this study, each point-scale *in-situ* measurement was assigned to the corresponding 0.25° grid cell based on its coordinates. When multiple measurements of either the same or different types were located within a single grid cell, their average was used as the representative grid value. Although this spatial aggregation approach can efficiently upscale point-scale observations to gridded data, it has inherent limitations in capturing SWE variability within heterogeneous terrain. Exploring higher resolution machine learning approaches and downscaling techniques remains an important and challenging task for improving the representation of sub-grid snow variability.

We trained the ML model to estimate SWE using a large collection of *in-situ* measurements primarily from the Northern Hemisphere. However, specific regions in the Northern Hemisphere, such as the Himalayas, Nepal, Japan, Central Asia, the Spanish Pyrenees, Iceland, and the land areas of Greenland, as well as areas in the Southern Hemisphere, including the Andes and Patagonia, lack *in-situ* measurements and were therefore not represented in the model training. In the case of Andes, although SWEML showed similar spatiotemporal characteristics to ADSWE through intercomparison, uncertainties remain regarding the reliability of the reference data in this region due to the absence of *in-situ* measurements. This limitation suggests that spatial gaps in *in-situ* measurements still remain. To address this issue, using bias-corrected region-specific remote sensing products or high-resolution model simulations (e.g., locally downscaled datasets) as additional training data will be an important and challenging next step for improving data accuracy in these regions.

### Guidance for data use

This study presents a new daily, gap-free global SWE product (SWEML) developed using a widely applied ML algorithm trained on *in-situ* SWE measurements, meteorological forcings, and terrain attributes. The performance of SWEML was consistently robust across regions and elevations when compared with reference SWE datasets. Notably, SWEML achieved the highest accuracy in snow-dominated and high-elevation regions. Moreover, SWEML improved the mitigation of regional biases compared with the reference datasets, partially addressing the limitations inherent in existing gridded reference products. It also captured temporal SWE features more effectively than the reference datasets, with marked improvements during the critical snowmelt period from spring to early summer. Comparisons with the high-resolution ADSWE and the spatially representative GAMMA further demonstrated that SWEML delivered reliable predictive performance in the Andes despite the absence of training data, and high accuracy across North America. Moreover, despite the challenge of learning temporal features from discontinuous training data, SWEML successfully reproduced interannual peak SWE patterns that were highly consistent with those of the reference datasets, demonstrating the robustness of this approach.

This study aimed to develop a new globally gap-free SWE product using *in-situ* measurements and ML, and to evaluate its performance against widely used reference SWE datasets. Our results demonstrate the capability of the RF model to capture the complexities of SWE dynamics, which remain difficult for conventional process-based models. Leveraging this capability, we developed a highly accurate global daily SWE product. Using observable predictors with the trained RF algorithm, SWE can be readily estimated on a global scale without explicit knowledge of the underlying physical processes. At the same time, this approach offers insights that may inform the development of physically based models, particularly for processes that are not yet well understood. Because ML algorithms learn SWE dynamics directly from input data, they have the potential to reveal important but previously unidentified processes. In the Andes, where no training data were available, SWEML nevertheless showed strong spatial and temporal consistency. This result highlights the ability of ML to overcome limitations such as the lack of training observations and demonstrates its potential to provide reliable estimates in data-sparse regions.

SWEML provides valuable information for snow-dominated regions and is therefore critical for managing water resources dependent on snowmelt. In particular, SWE anomaly analyses based on SWEML can be used to assess regional variations in snow in the context of climate change and the global water cycle, enabling the evaluation of hydrological impacts such as droughts and floods driven by snow variability. The SWEML dataset can also be used across various other applications, including improving the initialization of land surface models and global climate frameworks. In global-scale modeling, ensuring spatiotemporal continuity of data is essential. Many available high-resolution gridded SWE products are limited to specific regions, such as CONUS, the western U.S., or the Andes, while coarse-resolution products offer hemispheric or global coverage but often suffer from spatial limitations or reduced accuracy. By contrast, SWEML provides 41 years of globally gap-free SWE data, making it a valuable resource for large-scale modeling analysis. Assimilating SWEML into land surface models can further enhance the representation of snow processes and improve estimates of water and energy fluxes in Earth system modeling. To the best of our knowledge, while previous ML-based SWE datasets have been primarily developed at regional scales, SWEML represents the first globally gap-free SWE product generated using ML. It is expected to be widely applicable for evaluating the accuracy of SWE estimates derived from other approaches, including advanced snow models, data assimilation outputs, and alternative ML techniques. Finally, because ERA5 is open access and regularly updated, SWEML can be readily extended to incorporate newly available data, ensuring its long-term relevance.

## Supplementary information

Supplement

## Data Availability

The SWEML is available on Zenodo^135^ at the following link: 10.5281/zenodo.16822772. The download includes a single file in NetCDF format (see Data Records for a description of the contents). Our SWEML data are freely available for reuse or modification with attribution.

## Code availability

The code used in this study is available at the following GitHub repository: https://github.com/krsmsuh/SWEML.
