# Supplementary material for: Global 0.25-degree gridded Snow water equivalent data derived from machine learning using in-situ measurements
Source: Sci Data. 2026 Mar 10;13:739. doi: 10.1038/s41597-026-06895-z (PMC13179342; doi:10.1038/s41597-026-06895-z)
Supplement: Supplementary file 1 — Supplement [file 41597_2026_6895_MOESM1_ESM.pdf]

Supplementary Materials for

Global 0.25-degree gridded Snow water equivalent data derived from machine learning using *in-situ* measurements

Authors

Jungho Seo[1,2], Mahdi Panahi[1], JiHyun Kim[3,4], Sayed Bateni[2,5], Yeonjoo Kim[1]

Affiliations

1. Department of Civil and Environmental Engineering, Yonsei University, Seoul, 03722, South Korea

2. Department of Civil, Environmental and Construction Engineering, and Water Resources Research Center, University of Hawaii at Manoa, Honolulu, HI, 96822, USA

3. Department of Geography, Kyung Hee University, Seoul, 02447, South Korea

4. Department of Climate-Social Science Convergence, Kyung Hee University, Seoul, 02447, South Korea

5. UNESCO-UNISA African Chair in Nanoscience and Nanotechnology College of Graduate Studies, University of South Africa, Muckleneuk Ridge, Pretoria 392, South Africa

Corresponding author(s): Yeonjoo Kim (yeonjoo.kim@yonsei.ac.kr)

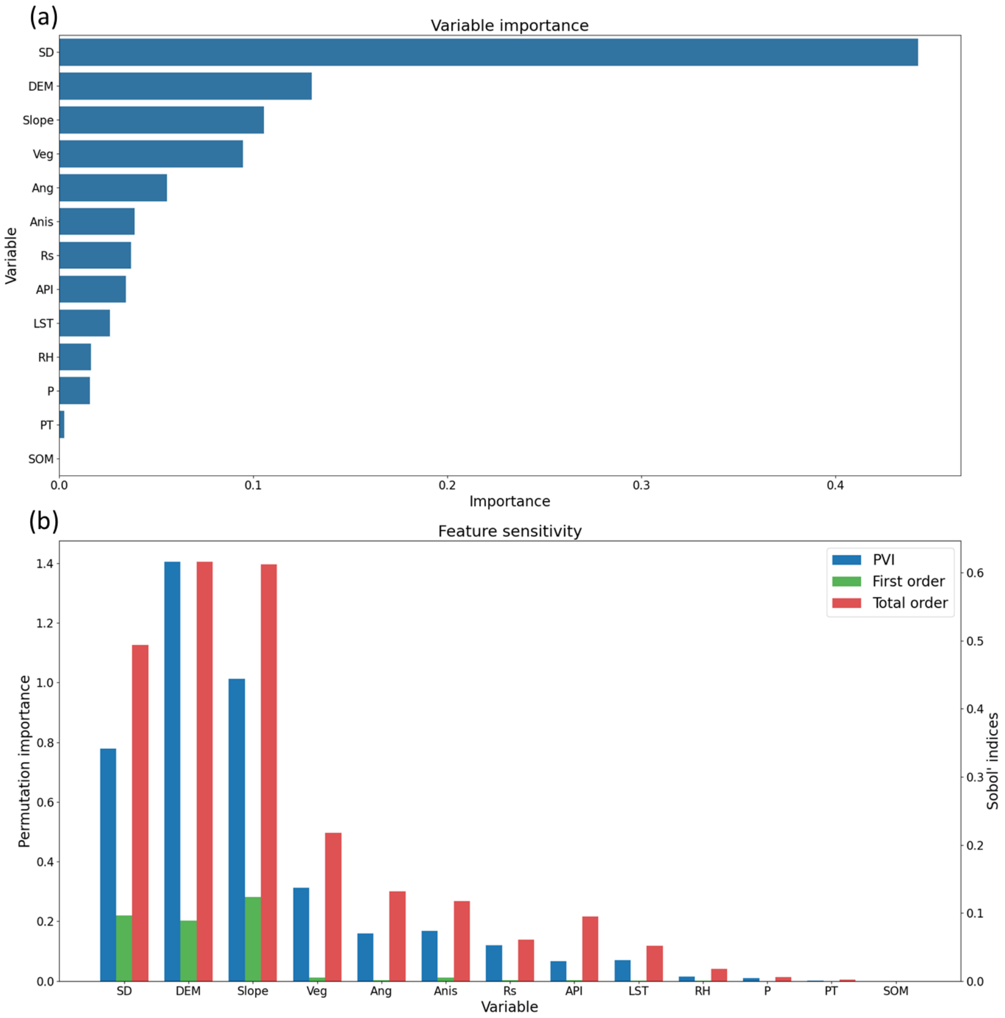

Figure S1: (a) Random forest-based variable importance and (b) feature sensitivity assessed using permutation variable importance (PVI) and Sobol sensitivity indices for the predictors used in SWE estimation. Variable importance indicates the relative contribution of each predictor to the overall model performance. PVI measures the reduction in prediction accuracy caused by the random shuffling of each predictor, while Sobol indices quantify the variance-based global sensitivity in terms of first order (independent) and total order (interaction) effects.

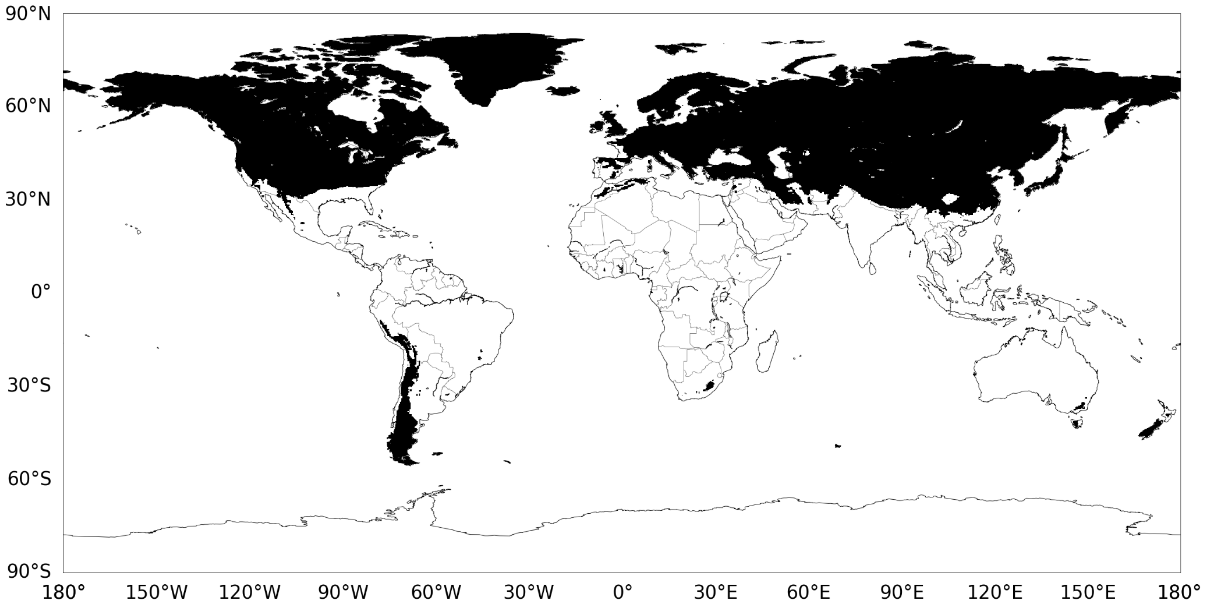

Figure S2: Snow observation mask indicating areas in black where snow was observed at least once during the study period.

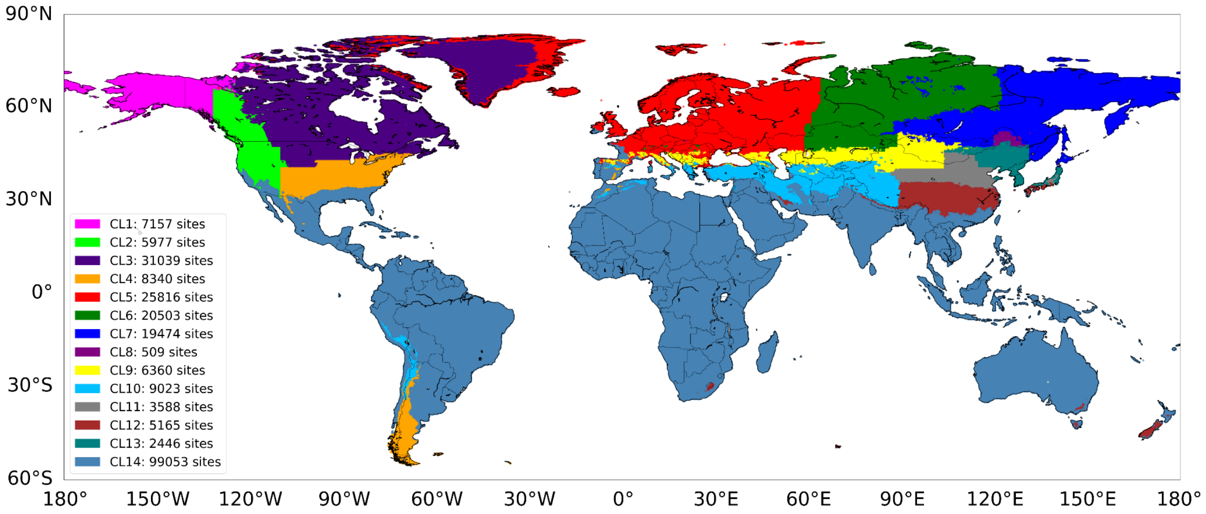

Figure S3. Results of the RFC, where all grid cells in the study domain were classified and assigned to cluster-specific RFR models.

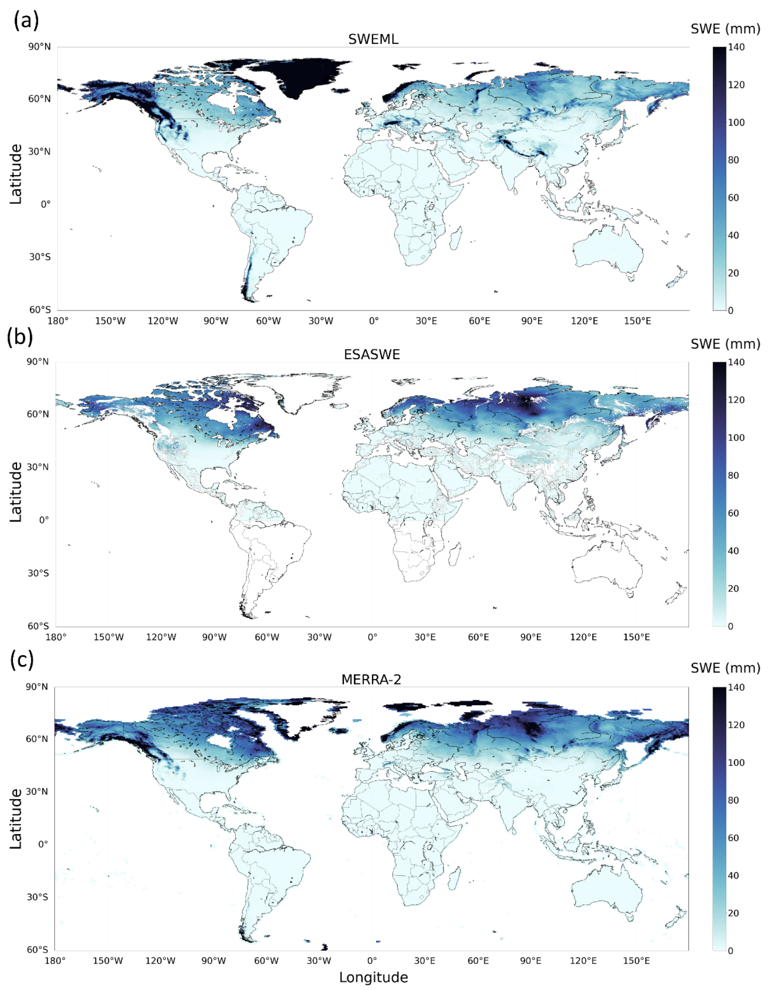

Figure S4: Annual mean SWE from (a) SWEML, (b) ESASWE, and (c) MERRA-2.

Table S1. Performance metrics for the 10-fold CV for each cluster. The values in parentheses represent the standard deviation between folds.

Model	Performance metric		
	RMSE	MAE	R-square
Cluster 1	78.5025 (1.2671)	30.2174 (0.4386)	0.9718 (0.0007)
Cluster 2	48.4342 (0.1559)	20.4617 (0.0472)	0.9295 (0.0005)
Cluster 3	23.8710 (0.7229)	10.9999 (0.0677)	0.9449 (0.0029)
Cluster 4	23.1797 (0.5882)	8.6578 (0.0684)	0.9397 (0.0002)
Cluster 5	39.6444 (0.6277)	20.7105 (0.2070)	0.9492 (0.0018)
Cluster 6	28.9940 (0.2273)	16.7558 (0.1166)	0.8552 (0.0021)
Cluster 7	9.7129 (0.1691)	4.5113 (0.0276)	0.8438 (0.0043)
Cluster 8	4.5726 (0.0176)	3.1330 (0.0105)	0.8960 (0.0010)
Cluster 9	12.4804 (0.0879)	4.8829 (0.0169)	0.8641 (0.0016)
Cluster 10	23.2518 (0.0830)	6.0978 (0.0126)	0.9836 (0.0001)
Cluster 11	4.2258 (0.0502)	1.9402 (0.0071)	0.7810 (0.0041)
Cluster 12	51.5473 (0.2388)	16.3612 (0.0374)	0.8400 (0.0014)
Cluster 13	6.8348 (0.0873)	3.6534 (0.0157)	0.7932 (0.0030)
Cluster 14	0.0496 (0.0359)	0.0016 (0.0007)	0.9812 (0.0006)

Table S2. Performance metrics for the LOSOCV for each cluster. The values in parentheses indicate the standard deviation between folds.

Model	Performance metric	
	RMSE	R-square
Cluster 1	28.745<br>(12.281)	0.699<br>(0.165)
Cluster 2	30.116<br>(11.241)	0.671<br>(0.154)
Cluster 3	23.895<br>(10.942)	0.543<br>(0.196)
Cluster 4	11.646<br>(10.270)	0.533<br>(0.165)
Cluster 5	25.672<br>(8.095)	0.677<br>(0.126)
Cluster 6	24.193<br>(9.865)	0.595<br>(0.129)
Cluster 7	11.676<br>(10.577)	0.697<br>(0.187)
Cluster 8	5.317<br>(2.367)	0.806<br>(0.139)
Cluster 9	5.496<br>(9.357)	0.549<br>(0.154)
Cluster 10	6.010<br>(9.106)	0.550<br>(0.146)
Cluster 11	2.631<br>(3.344)	0.555<br>(0.141)
Cluster 12	14.141<br>(12.054)	0.524<br>(0.137)
Cluster 13	5.590<br>(5.266)	0.670<br>(0.149)
Cluster 14	0.138<br>(0.091)	0.598<br>(0.224)

Table S3. Performance metrics of the RFC assessed using stratified 10-fold cross validation.

Performance metric	Total average	Standard deviation
Accuracy	0.895	0.0006
Precision (weighted)	0.919	0.0004
Recall (weighted)	0.895	0.0006
F1-score (weighted)	0.898	0.0006
ROC-AUC (one-vs-rest)	0.992	0.0001
